# Machine learning based predictive model for cardiovascular disease mortality risk in patients with stage 4 cardiovascular–kidney–metabolic syndrome

**DOI:** 10.1097/MD.0000000000051266

**Published:** 2026-10-23

**Authors:** Pengyu Zhang, Sining Wang, He Yang, Yixin He, Zhihui Xu, Taijin Wu, Zhouyu Su, Weizhe Deng, Qiang Zhang

**Affiliations:** a Department of Nephrology and Rheumatology, Tongde Hospital of Zhejiang Province, Hangzhou, China; b Department of Nephrology, The Second Affiliated Hospital of Harbin Medical University, Harbin, China; c Department of Nephrology, The 962nd Hospital of the PLA, Harbin, China; d Department of Cardiology, The First Affiliated Hospital of Hainan Medical University, Haikou, China; e Department of Rheumatology and Chinese Medicine, The 962nd Hospital of the PLA, Harbin, China; f Department of Rheumatology and Immunology, The Second Affiliated Hospital of Zhejiang Chinese Medical University, Hangzhou, China.

**Keywords:** cardiovascular disease mortality, cardiovascular–kidney–metabolic, chronic kidney disease, machine learning, SHapley Additive exPlanations

## Abstract

Patients with stage 4 cardiovascular–kidney–metabolic (CKM) syndrome are exposed to an elevated cardiovascular disease (CVD) mortality risk. However, large-sample, multidimensional prediction models for CVD mortality risk that integrate clinical indicators, as well as highly applicable supporting tools, remain lacking. A total of 2893 patients with stage 4 CKM were randomly allocated to training and validation sets at a 7:3 ratio. The prediction model was constructed via variable screening using least absolute shrinkage and selection operator combined with multivariate Cox regression. Discrimination, calibration, and clinical utility of the model were evaluated with receiver operating characteristic curves, calibration plots, and decision curve analysis. A nomogram and web-based calculator were constructed. Six machine learning models were used to validate the model, and predictive factors were identified via SHapley Additive exPlanations analysis. Nine risk factors were ultimately identified for constructing the predictive model: age, gender, mobility difficulty, chronic kidney disease, anemia, neutrophil, blood urea nitrogen, albumin, and C-reactive protein. The model’s discriminate ability on training set (C-index = 0.773) was comparable that on validation set (C-index = 0.779). Additionally, the model exhibited optimal performance in predicting 15-year CVD mortality (area under the curve = 0.871). The calibration curve demonstrated comparable fit, and decision curve analysis revealed that the nomogram score provided higher clinical benefit compared with any single variable. Participants with high nomogram scores exhibited a lower survival rate (log-rank test, *P* < .001). Among the 6 machine learning models, Random Forest and XGBoost achieved the best performance. SHapley Additive exPlanations analysis further indicated that age and C-reactive protein made the greatest contributions to predicting CVD mortality risk. The CVD mortality risk prediction model developed for patients with stage 4 CKM demonstrated robust performance. This model may enable clinicians to identify high-risk patients early and to formulate individualized intervention strategies through a reliable decision support tool.

## 1. Introduction

Cardiovascular–kidney–metabolic (CKM) syndrome is a multisystem disorder characterized by interactions among the cardiovascular disease (CVD), renal, and endocrine metabolic systems.^[1,2]^ Approximately 1/3 of elderly individuals enrolled in Medicare service in the US have chronic kidney disease (CKD), congestive heart failure (CHF), or diabetes; however, these patients account for over 1/2 of expenditures.^[3]^ The combined prevalence of CKM conditions and their associated healthcare utilization are projected to increase, thereby placing substantial pressure on healthcare systems.^[4]^

CKM syndrome is defined by a shared pathophysiological basis, encompassing metabolic disturbances, obesity, hyperlipidemia, hyperglycemia, hypertension, CKD, and CVD. This constellation of features ultimately results in the progressive deterioration of multiple organ function.^[5,6]^ These factors synergistically contribute to a significant increase in the morbidity and mortality associated with CKD and CVD.^[7]^ In stage 4 CKM syndrome, patients typically exhibit significant renal function impairment, including tubular dysfunction or reduced glomerular filtration rate and metabolic abnormalities, such as severe insulin resistance, decreased glucose tolerance, elevated triglycerides, and low high-density lipoprotein cholesterol, alongside arteriosclerosis and cardiac structural changes as CVD risk factors.^[8,9]^ This constellation of abnormalities results in a significantly elevated risk of CVD mortality, which is substantially higher than the risk observed in stages 0 to 3.^[10]^ Accurately predicting the risk of CVD mortality in patients with stage 4 CKM is therefore critical for guiding individualized treatment strategies.^[11,12]^ Reduced renal function, impaired nutritional status, and elevated inflammatory markers have been proposed as potential predictors of CVD mortality risk.^[13]^

Research on CKM has emerged as a prominent area of investigation in recent years, with a growing body of related literature. Notably, large cohort studies such as the China Health and Retirement Longitudinal Study and the National Health and Nutrition Examination Survey (NHANES), along with correlation and survival analyses, have provided novel insights into the early identification of CKM, disease outcomes, and intervention guidance.^[14,15]^ With the advancement of interdisciplinary collaboration between clinical medicine and computer science, predictive models are increasingly being developed and implemented in clinical practice. These models integrate dimensional clinical data for risk assessment, thereby enhancing the accuracy and clinical utility of predictions.^[16]^ Machine learning algorithms offer the advantage of greater accuracy compared with single models in outcome classification, risk prediction, and survival analysis.^[17]^ By searching Pubmed and Web of Science literature, we found that there is currently a lack of community surveys of CVD mortality risk among CKM community participants in large samples and a well-developed mortality risk assessment model.

Our study aims to develop and validate a multiple-parameter model for predicting CVD mortality risk in patients with stage 4 CKM. This model will be developed using data from the US-based NHANES database and machine learning algorithms, with the goal of providing clinicians with a decision tool to optimize the assessment of patients’ survival prognosis.

## 2. Materials and methods

### 2.1. Data source and study population

Data were collected from the NHANES, conducted by the National Center for Health Statistics (NCHS) of the Centers for Disease Control and Prevention. A complex, stratified, multistage probability sampling design was employed to evaluate the health and nutritional status of the civilian, non-institutionalized US population. Comprehensive data, including demographic characteristics, medical history, lifestyle behaviors, and health-related quality of life, were systematically obtained via standardized questionnaires, physical examinations, and laboratory assessments. The protocol was approved by the NCHS Research Ethics Review Board, and written informed consent was obtained from all participants. Public use datasets were downloaded free of charge from the official NHANES repository (https://www.cdc.gov/nchs/nhanes).

Ten consecutive 2-year cycles of NHANES (1999–2018) were merged to create the analytical dataset. After initial pooling, 1,01,316 participants were screened. Rigorous exclusion criteria were subsequently applied to minimize selection bias. In total, 98,423 individuals were excluded: 46,235 aged < 20 years, 1541 who were pregnant, 29,026 with incomplete CKM data, and 21,552 who did not meet stage 4 CKM criteria. An additional 70 participants without mortality linkage were removed, leaving 2893 eligible subjects for the final analysis. Participant inclusion, grouping, and study design were shown in the flowchart (Fig. 1).

**Figure 1.**
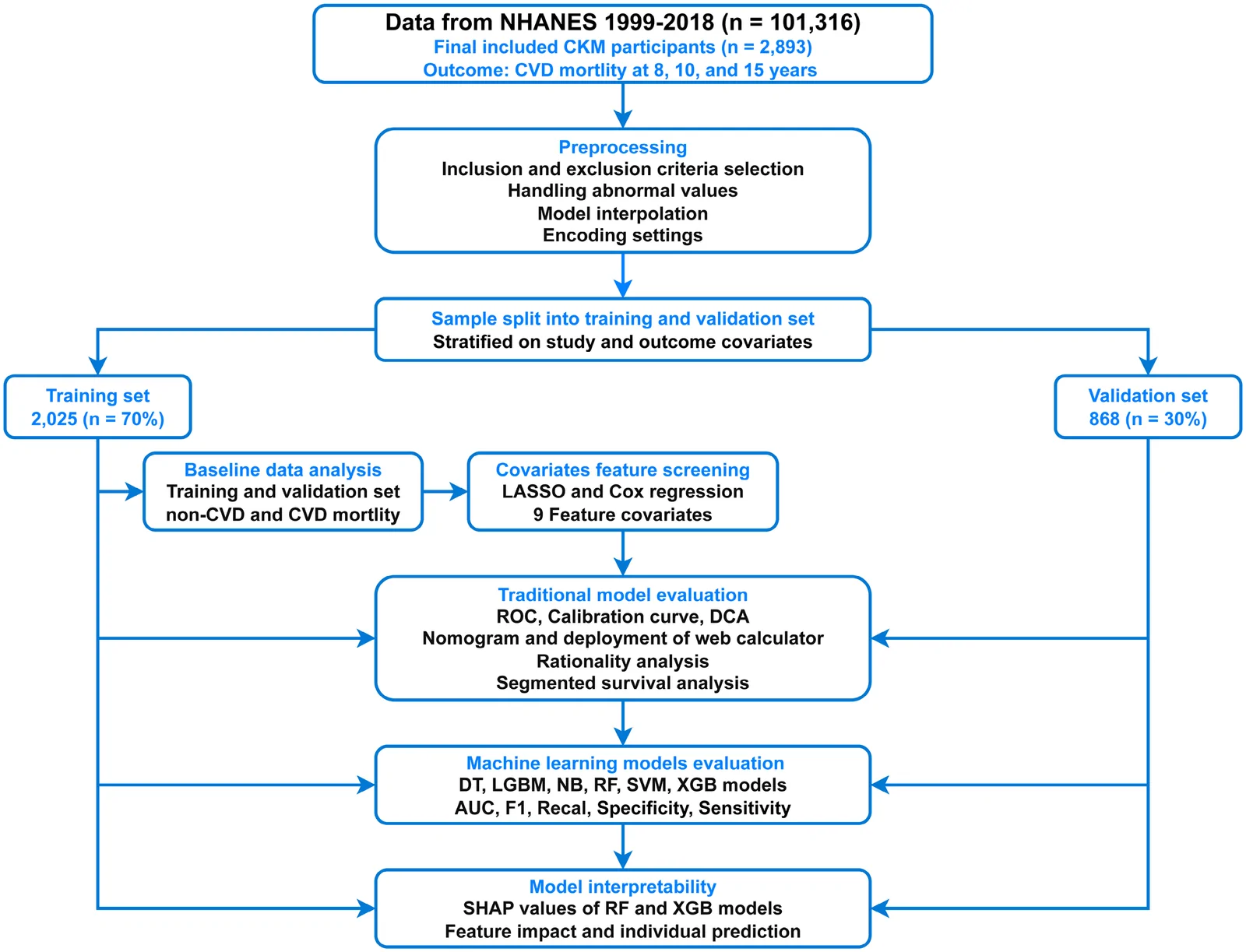
Flowchart of screening, grouping, and research design. AUC = area under the curve, CKM = cardiovascular-kidney-metabolic, CVD = cardiovascular disease, DCA = decision curve analysis, DT = decision tree, LASSO = least absolute shrinkage and selection operator, LGBM = light gradient boosting machine, NB = Naive Bayes, NHANES = National Health and Nutrition Examination Survey, RF = Random Forest, ROC = receiver operating characteristic, SHAP = SHapley Additive exPlanations, SVM = support vector machine, XGB = extreme gradient boosting.

### 2.2. Definition of cardiovascular–kidney–metabolic syndrome

Classification criteria for CKM syndrome were delineated in the 2023 American Heart Association scientific statement.^[18]^ The framework proposes a continuum of 5 stages (0–4), with stage 1 representing excess adiposity, stage 2 encompassing metabolic or renal risk factors (diabetes, CKD, hypertension, dyslipidemia), stage 3 subclinical CVD disease, and stage 4 overt CVD.^[19]^ Stage 4 is defined by the concurrent presence of chronic kidney disease, metabolic abnormalities (hypertension, diabetes mellitus, dyslipidemia, or metabolic syndrome), and clinical CVD disease. CKD was operationalized as an estimated glomerular filtration rate (eGFR) < 60 mL/min/1.73 m^2^ or a urinary albumin-to-creatinine ratio > 30 mg/g, with eGFR calculated using the CKD-EPI creatinine equation.^[20,21]^ Metabolic syndrome was diagnosed when central obesity coexisted with at least two of the following 4 criteria: hypertriglyceridemia, low-density lipoprotein cholesterol, hypertension, or hyperglycemia.^[22]^ CVD ascertainment was based on self-reported physician-diagnosed CHF, coronary heart disease (CHD), angina, myocardial infarction (MI), and stroke, obtained from standardized questionnaires.^[23]^

### 2.3. Definition of cardiovascular disease mortality

Death events were determined through the longitudinal follow-up data of death events with NHANES as of December 31, 2019, for all causes of mortality.^[24]^ CVD mortality was defined as an underlying cause of death assigned to ICD-10 codes I00–I11, I13, I20–I51 or I60–I69, denoting diseases of the circulatory system.^[25]^

### 2.4. Covariates

Demographic and lifestyle covariates comprised age, sex, drink status, and smoke status. Data on weight, height, and blood pressure were measured during physical examinations at mobile medical units. Blood and urine specimens were measured at the NHANES mobile examination centers. Hypertension was defined as a self-reported diagnosis or current antihypertensive medication use, or a mean systolic blood pressure ≥ 130 mm Hg or diastolic blood pressure ≥ 85 mm Hg on examination. Diabetes mellitus was recorded if participants reported a physician diagnosis, or fasting plasma glucose ≥ 7.0 mmol/L or 2-hour oral glucose tolerance test ≥ 11.1 mmol/L. Metabolic syndrome was present when ≥3 of the following 5 criteria were met: waist circumference ≥ 90 cm (male) or ≥85 cm (female), triglycerides ≥ 1.7 mmol/L, HDL < 1.0 mmol/L (male) or < 1.3 mmol/L (female), blood pressure ≥ 130/85 mm Hg, fasting glucose ≥ 5.5 mmol/L. Anemia was defined as hemoglobin < 13 g/dL (male) or <12 g/dL (female). Self-reported asthma, chronic obstructive pulmonary disease, cancer, sleep disorder, mobility difficulty (no difficulty, some difficulty, much difficulty, unable) and CHD family history were extracted from questionnaire data.

### 2.5. Statistical analysis

Continuous variables were summarized as M (P25, P75), and categorical variables as counts (%). Group differences were evaluated with Chi-squared test, Wilcoxon rank sum, or Mann–Whitney *U* test as appropriate. Missing values were imputed by means of the Random Forest (RF) algorithm; variables with >30% missingness were excluded. Variable missing rates were presented in Table S1, Supplemental Digital Content 1. The cohort was randomly allocated 7:3 to training and validation sets. Model derivation comprised variable screening using least absolute shrinkage and selection operator regression, followed by multivariable Cox proportional hazards modeling with backward elimination guided by both Akaike information criteria and Bayesian information criteria. Model discrimination, calibration, and clinical utility were quantified with receiver operating characteristic analysis, calibration plots, and decision curve analysis, respectively. A nomogram was constructed, internal plausibility was examined, and KM survival curves were generated across nomogram-derived risk tertiles. 6 machine learning algorithms were trained to predict CVD mortality, and feature importance in the best performing model was evaluated using SHapley Additive exPlanations (SHAP) values. Analyses and visualizations were conducted with R (v4.4.2; R Foundation for Statistical Computing, Austria), and PCPM (v5.46.5; Jingding Statistics, China). *P* < .05 was considered statistically significant.

## 3. Results

### 3.1. Baseline characteristics of participants

The median age of participants with stage 4 CKM syndrome was 69 years. 1619 (55.96 %) were male, 2077 (71.79 %) had hypertension, 1651 (57.07 %) met criteria for metabolic syndrome, and 1351 (46.70 %) reported a CHD family history. Abdominal obesity was present in 68.82%, hyperglycemia in 56.48%, high alcohol consumption in 58.31 % and smoking in 59.97 %. Individuals who experienced CVD mortality differed significantly from those alive or with non-CVD mortality with respect to age, sex, BMI, blood pressure, sleep disorder, mobility difficulty, CHF, MI, CKD and CKD stage, asthma, anemia, lymphocyte, monocyte and neutrophil (NEUT), red cell count, urinary albumin-to-creatinine ratio, urinary protein, blood urea nitrogen (BUN), uric acid (UA), serum creatinine, eGFR, alanine aminotransferase, globulin, calcium, potassium, and vitamin D (*P* < .05; Table 1). No statistically significant differences were observed between the training and validation sets (*P* > .05; Table S2, Supplemental Digital Content 2). Group comparisons for the training set and validation set were provided in supplementary materials (Table S3, Supplemental Digital Content 3 and Table S4, Supplemental Digital Content 4).

**Table 1:** Baseline characteristics of participants with stage 4 of CKM.

Characteristics	Total	Alive and no-CVD mortality	CVD mortality	*P* value
	n = 2,893	n = 2,234	n = 659	
Age, M (P25, P75)	69.00 (60.00, 78.00)	66.00 (58.00, 76.00)	75.00 (68.00, 80.00)	<.001
Gender, n (%)				.002
Male	1619 (55.96%)	1216 (54.43%)	403 (61.15%)	
Female	1274 (44.04%)	1018 (45.57%)	256 (38.85%)	
BMI, M (P25, P75)	29.00 (25.40, 33.37)	29.14 (25.60, 33.70)	28.40 (24.81, 32.20)	<.001
SBP Avg, M (P25, P75)	130.67 (117.33, 146.00)	129.33 (116.67, 144.00)	134.85 (120.00, 153.33)	<.001
DBP Avg, M (P25, P75)	68.00 (60.00, 76.00)	68.00 (60.67, 76.67)	66.00 (58.67, 73.33)	<.001
Drink, n (%)	1687 (58.31%)	1284 (57.48%)	403 (61.15%)	.092
Smoke, n (%)	1735 (59.97%)	1353 (60.56%)	382 (57.97%)	.232
Sleep disorder, n (%)	1090 (37.68%)	887 (39.70%)	203 (30.80%)	<.001
CHD family history, n (%)	628 (21.71%)	492 (22.02%)	136 (20.64%)	.448
Mobility difficult, n (%)				<.001
No difficult	1944 (67.20%)	1535 (68.71%)	409 (62.06%)	
Some difficult	550 (19.01%)	422 (18.89%)	128 (19.42%)	
Much difficult	237 (8.19%)	175 (7.83%)	62 (9.41%)	
Unable	162 (5.60%)	102 (4.57%)	60 (9.10%)	
Complications, n (%)				
Hypertension	2077 (71.79%)	1594 (71.35%)	483 (73.29%)	.331
Diabetes	1244 (43.00%)	946 (42.35%)	298 (45.22%)	.190
CHF	873 (30.18%)	638 (28.56%)	235 (35.66%)	<.001
CHD	1088 (37.61%)	843 (37.74%)	245 (37.18%)	.795
Angina	734 (25.37%)	552 (24.71%)	182 (27.62%)	.132
MI	1159 (40.06%)	867 (38.81%)	292 (44.31%)	.011
Stroke	996 (34.43%)	764 (34.20%)	232 (35.20%)	.633
CKD	1351 (46.70%)	904 (40.47%)	447 (67.83%)	<.001
Asthma	556 (19.22%)	453 (20.28%)	103 (15.63%)	.008
COPD	222 (7.67%)	165 (7.39%)	57 (8.65%)	.284
Anemia	473 (16.35%)	333 (14.91%)	140 (21.24%)	<.001
Cancer	589 (20.36%)	450 (20.14%)	139 (21.09%)	.595
High GLU	1634 (56.48%)	1251 (56.00%)	383 (58.12%)	.335
Low HDL	937 (32.39%)	737 (32.99%)	200 (30.35%)	.203
High TG	972 (33.60%)	744 (33.30%)	228 (34.60%)	.536
Abdominal obesity	1991 (68.82%)	1554 (69.56%)	437 (66.31%)	.114
MetS	1651 (57.07%)	1267 (56.71%)	384 (58.27%)	.478
CKD stage, n (%)				<.001
Stage I	168 (5.81%)	122 (5.46%)	46 (6.98%)	
Stage II	314 (10.85%)	214 (9.58%)	100 (15.17%)	
Stage III	720 (24.89%)	480 (21.49%)	240 (36.42%)	
Stage IV	81 (2.80%)	46 (2.06%)	35 (5.31%)	
Stage V	31 (1.07%)	20 (0.90%)	11 (1.67%)	
Laboratory examinations, M (P25, P75)				
WBC, 10^3^/μL	6.90 (5.70, 8.20)	6.90 (5.70, 8.20)	6.90 (5.70, 8.30)	.542
LYM, 10^3^/μL	1.80 (1.40, 2.30)	1.83 (1.50, 2.30)	1.70 (1.30, 2.10)	<.001
MONO, 10^3^/μL	0.60 (0.50, 0.70)	0.60 (0.50, 0.70)	0.60 (0.50, 0.70)	.022
NEUT, 10^3^/μL	4.20 (3.20, 5.20)	4.10 (3.20, 5.10)	4.30 (3.40, 5.50)	.006
PLT, 10^3^/μL	223.00 (187.00, 267.92)	225.00 (189.00, 268.00)	220.00 (184.00, 267.00)	.206
RBC, 10^6^/μL	4.58 (4.23, 4.93)	4.59 (4.26, 4.93)	4.53 (4.11, 4.93)	.002
Hb, g/dL	13.95 (12.90, 15.00)	14.00 (12.90, 15.00)	13.89 (12.70, 15.00)	.061
UACR, mg/g	14.29 (6.62, 46.89)	12.32 (6.17, 35.87)	25.51 (9.50, 83.31)	<.001
UPRO, μg/mL	15.60 (6.60, 48.76)	13.80 (6.20, 39.63)	25.60 (8.70, 88.40)	<.001
BUN, mg/dL	16.00 (12.00, 21.00)	15.10 (12.00, 20.00)	18.00 (14.00, 24.00)	<.001
UA, mg/dL	5.94 (5.00, 7.00)	5.90 (4.90, 6.90)	6.10 (5.20, 7.30)	<.001
Scr, mg/dL	1.00 (0.80, 1.20)	0.97 (0.80, 1.19)	1.09 (0.90, 1.38)	<.001
eGFR, ml/min	70.66 (53.52, 88.17)	73.95 (57.48, 89.96)	59.01 (43.79, 78.90)	<.001
ALB, mg/dL	4.10 (3.90, 4.30)	4.10 (3.90, 4.30)	4.10 (3.90, 4.30)	.394
ALT, U/L	20.00 (16.00, 26.00)	20.89 (16.00, 27.00)	18.00 (15.00, 24.00)	<.001
AST, U/L	23.00 (19.00, 27.53)	23.00 (19.00, 28.00)	23.00 (19.00, 27.00)	.800
GLB, U/L	3.00 (2.70, 3.30)	3.00 (2.70, 3.30)	3.10 (2.80, 3.40)	.003
GLU, mg/dL	103.00 (93.00, 123.00)	103.00 (93.00, 122.00)	104.00 (93.00, 126.00)	.128
HbA1c, %	5.80 (5.50, 6.40)	5.80 (5.40, 6.40)	5.90 (5.50, 6.50)	.121
Ca, mmol/L	2.35 (2.28, 2.40)	2.35 (2.28, 2.40)	2.35 (2.30, 2.43)	.033
Fe, mmol/L	79.00 (62.00, 99.00)	79.00 (62.00, 100.00)	77.00 (61.00, 98.00)	.118
Na, mmol/L	139.55 (138.00, 141.00)	139.70 (138.00, 141.00)	139.10 (138.00, 141.00)	.058
K, mmol/L	4.10 (3.90, 4.33)	4.10 (3.90, 4.30)	4.19 (3.90, 4.40)	<.001
TC, mg/dL	179.00 (154.00, 209.00)	178.00 (153.69, 208.56)	179.20 (156.00, 212.00)	.310
TG, mg/dL	125.00 (89.00, 173.00)	123.93 (87.00, 172.00)	127.00 (95.00, 176.00)	.107
LDL-C, mg/dL	99.03 (79.00, 124.00)	100.00 (79.00, 124.00)	99.00 (79.00, 124.00)	.598
HDL-C, mg/dL	48.00 (40.00, 58.00)	48.00 (40.00, 58.00)	48.00 (40.00, 59.00)	.618
CRP, mg/dL	0.32 (0.17, 0.58)	0.33 (0.18, 0.57)	0.30 (0.16, 0.64)	.379
VitD, nmol/L	61.10 (45.90, 77.90)	61.60 (46.10, 79.50)	59.10 (44.90, 73.46)	.004

*P* < .05 indicates a significant difference.

ALB = albumin, ALT = alanine aminotransferase, AST = aspartate aminotransferase, BMI = body mass index, BUN = blood urea nitrogen, Ca = Calcium, CHD = coronary heart disease, CHF = congestive heart failure, CKD = chronic kidney disease, CKM = cardiovascular-kidney-metabolic, COPD = chronic obstructive pulmonary disease, CRP = C-reactive protein, CVD = cardiovascular disease, DBP Avg = diastolic blood pressure average, eGFR = estimated glomerular filtration rate, Fe = Iron, GLB = globulin, GLU = glucose, Hb = hemoglobin, HbA1c = glycated hemoglobin, HDL-C = high-density lipoprotein cholesterol, K = potassium, LDL-C = low-density lipoprotein cholesterol, LYM = lymphocyte, Mets = metabolic syndrome, MI = myocardial infarction, MONO = monocyte, Na = Sodium, NEUT = neutrophil, PLT = platelet, RBC = red blood cell, SBP Avg = systolic blood pressure average, Scr = serum creatinine, TC = total cholesterol, TG = triglyceride, UA = uric acid, UACR = urinary albumin creatinine ratio, UPRO = urinary protein, VitD = vitamin D, WBC = white blood cell.

### 3.2. Model specification

Least absolute shrinkage and selection operator regression on the training set screened out 8 variables (age, CKD, mobility difficulty, anemia, NEUT, BUN, albumin [ALB], and C-reactive protein [CRP]; Fig. 2). By default, we included 2 variables, age and gender, in the model to construct a multivariate COX stepwise regression, and all of them were selected. Ultimately, 9 variables were included to construct the model (AIC = 5895.577, Bayesian information criteria = 5957.323). CKD (HR = 1.702, 95% CI: 1.373–2.110), mobility difficulty (HR = 1.812, 95% CI: 1.291–2.545), and ALB (HR = 0.584, 95% CI: 0.432–0.789; Table 2).

**Table 2:** Backward stepwise multiple Cox regression on training set.

Characteristic	CKM	CVD mortality	HR	95% CI	*P* value	VIF
Age	2025	459	1.066	1.055–1.078	<.001	1.082
Gender						1.081
Male	1127	275	-	-		
Female	898	184	0.724	0.597–0.879	.001	
Mobility difficult						1.091
No difficult	1371	290	-	-		
Some difficult	370	87	1.256	0.982–1.606	.070	
Much difficult	173	42	1.395	1.002–1.941	.049	
Unable	111	40	1.812	1.291–2.545	.001	
CKD						1.183
No	963	311	-	-		
Yes	1062	148	1.702	1.373–2.110	<.001	
Anemia						1.202
No	1697	359	-	-		
Yes	328	100	1.239	0.970–1.583	.087	
NEUT	2025	459	1.075	1.010–1.144	.024	1.217
BUN	2025	459	1.023	1.013–1.033	<.001	1.233
ALB	2025	459	0.584	0.432–0.789	<.001	1.171
CRP	2025	459	1.108	1.031–1.192	.005	1.012

*P* < .05 indicates a significant difference.

ALB = albumin, BUN = blood urea nitrogen, CKD = chronic kidney disease, CKM = cardiovascular–kidney–metabolic, CRP = C-reactive protein, CVD = cardiovascular disease, NEUT = neutrophil. CI = confidence interval, HR = hazard ratio, VIF = variance inflation factor.

**Figure 2.**
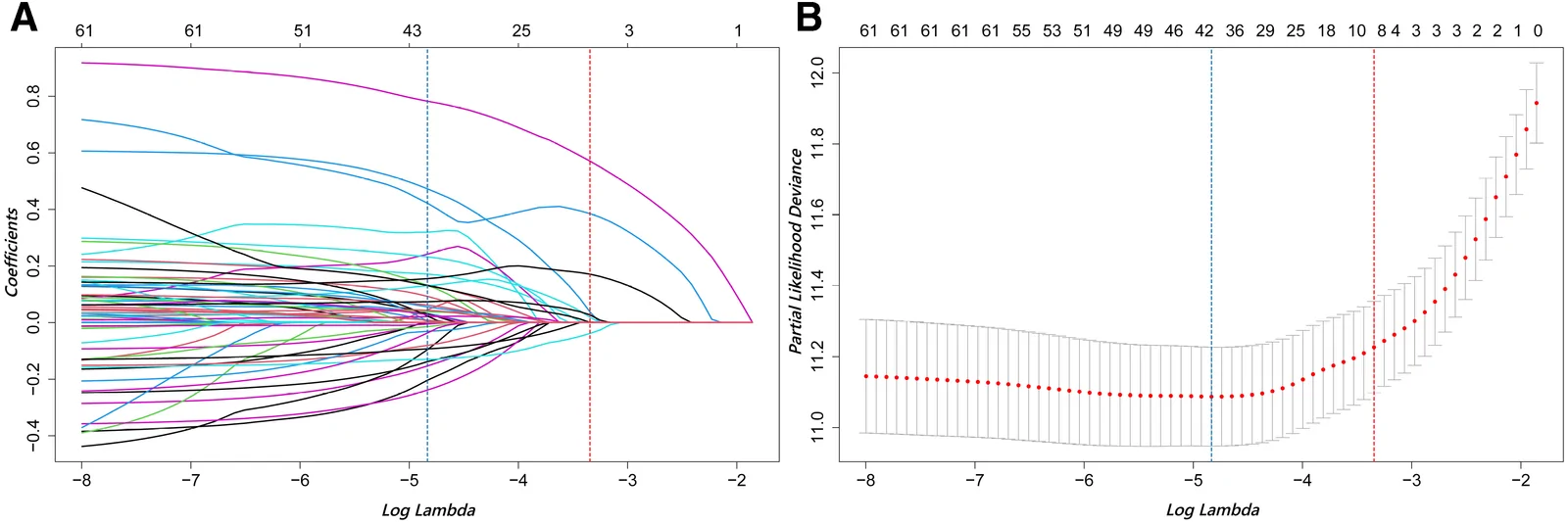
LASSO regression feature selection on training set. The trend of each feature coefficient in LASSO regression with the change of the logarithmic regularization parameter Lambda (A), and the range of the logarithmic regularization parameter λ in LASSO regression for the binomial deviation used to determine the model fitting effect. 61 variables were included in the screening; Lambda min screened out 40 related variables, and Lambda Se screened out 8 related variables (B). LASSO = least absolute shrinkage and selection operator.

### 3.3. Model evaluation

The discrimination and stability of the model in predicting CVD mortality at 8 years, 10 years, and 15 years were evaluated by drawing receiver operating characteristic and calibration curves. The training set conformity index (C-index) = 0.773 (0.751–0.795) was stable and good after 500 bootstrap (bias = 0.002, std.error = 0.010) sampling. The prediction of 15-year CVD mortality was best (area under the curve [AUC] = 0.849, 95% CI: 0.815–0.883). The validation set obtained a similar C-index = 0.779 (0.740–0.808) and was stable and good after 500 bootstrap (bias = 0.004, std.error = 0.018) sampling. The prediction of 15-year CVD mortality was best (AUC = 0.871, 95% CI: 0.826–0.917). The training set 8-year Brier score (BS) = 0.121, 10-year (BS = 0.140), and 15-year (BS = 0.140) and validation set 8-year (BS = 0.130) time point data for 10 years (BS = 0.151) and 15 years (BS = 0.180) were scattered near the diagonal, and the error line endpoints were distributed on either side of the diagonal, suggesting that the overall survival data were consistent with the actual observed data (Fig. 3).

**Figure 3.**
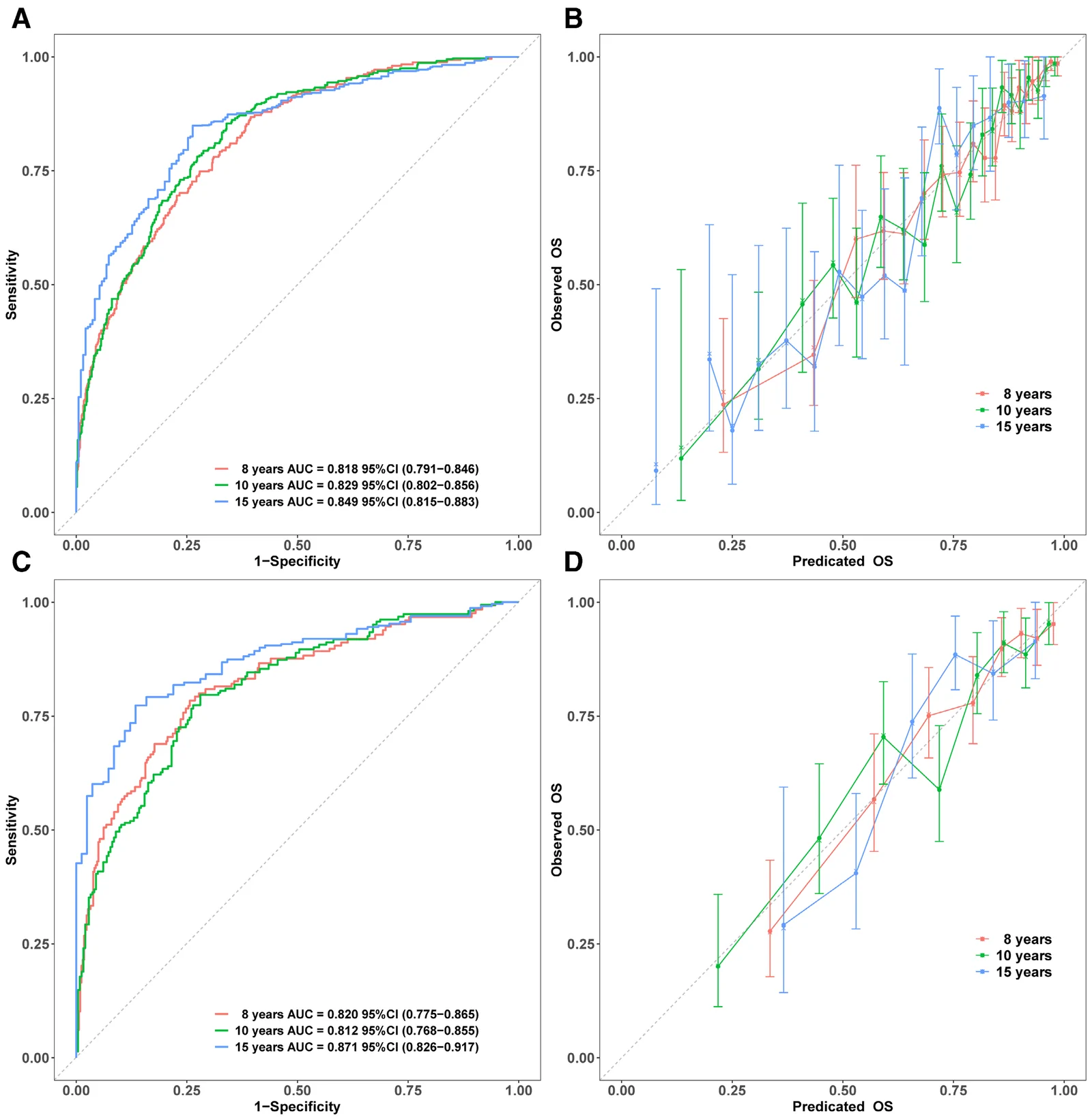
Model evaluation for the training and validation sets. ROC of the training set based on the logistic regression model (A), the calibration curve on training set (B), the ROC on validation set (C), and the calibration curve on validation set (D), with curves of different colors representing different prediction times. AUC = area under the curve, CI = confidence interval, ROC = receiver operating characteristic.

### 3.4. Deployment of nomogram and web-based calculator

We constructed a nomogram for predicting the risk of CVD mortality in CKM syndrome patients based on COX model regression coefficients and cutoff values, showing the relationship between variables and corresponding scores and the probability of CVD mortality. In CKM patients, the risk of CVD mortality increased with age, NEUT, BUN, and CRP. A decrease in ALB increases the risk of CVD mortality. Male, mobility-impaired, CKD, and anemic participants had an increased risk of CVD mortality. Nomograms also showed the total score, risk stratification, and survival pro mapping relationships and risk intervals (Fig. 4). Web calculator address: https://zpy572551000.shinyapps.io/dynnomapp/. The operation page was displayed in Figure S1, Supplemental Digital Content 5.

**Figure 4.**
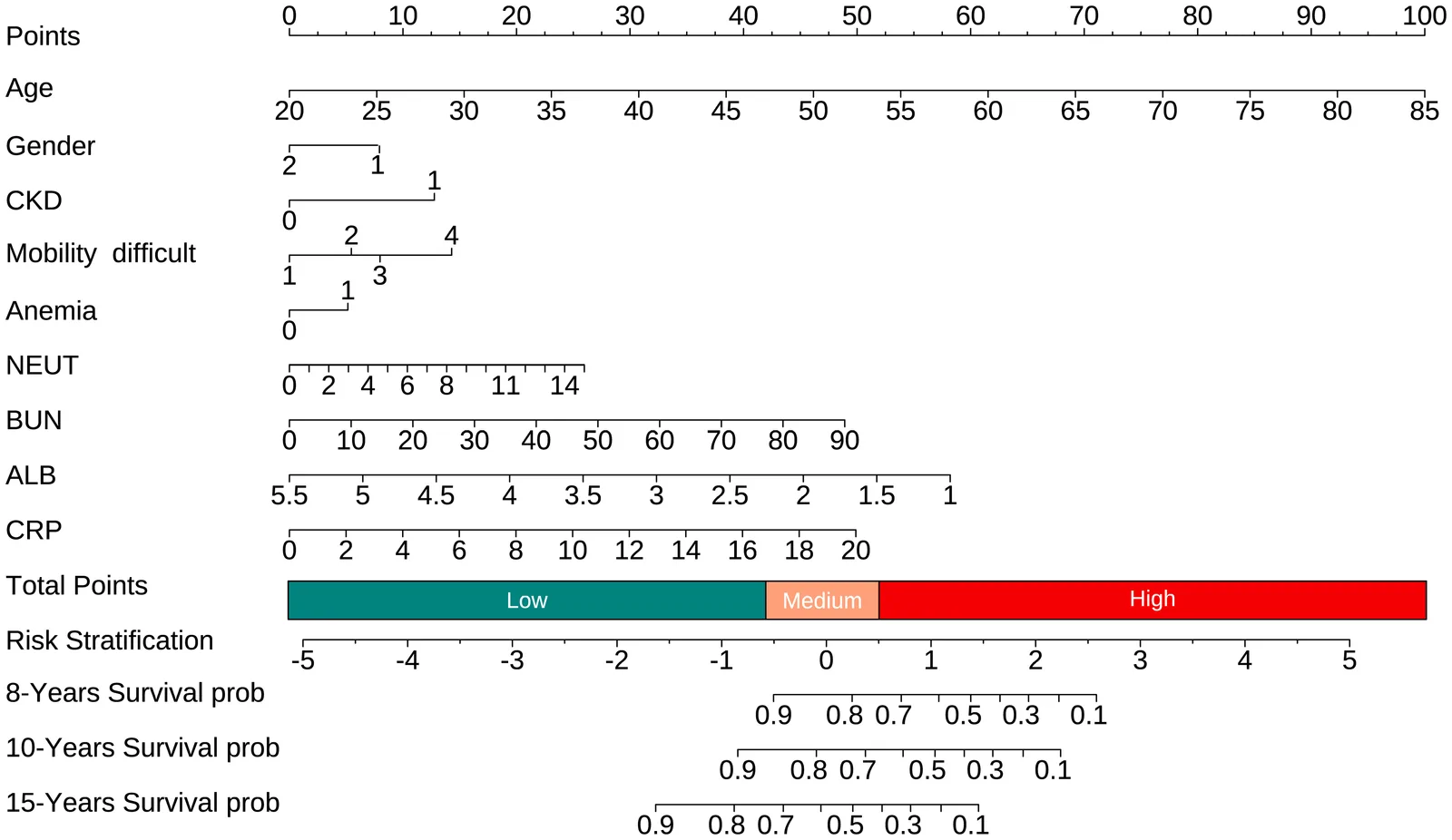
Nomogram and score segments of the training set. Display covariates: gender (1 = male, 2 = female), CKD: chronic kidney disease (0 = no, 1 = yes) mobility difficult(1 = no difficult, 2 = some difficult, 3 = much difficult, 4 = unable), anemia (0 = no, 1 = yes), Total points were represented by green, orange, and red from the lowest to the highest tertile. ALB = albumin, BUN = blood urea nitrogen, CKD = chronic kidney disease, CRP = C-reactive protein, NEUT = neutrophil.

Nomogram score formula = age (1.538 × age − 30.769) + gender (male = 11.057, female = 0) + mobility difficult (no difficult = 0, some difficult = 7.619, much difficult = 7.075, unable = 19.703) + CKD (no = 0, yes = 16.119) + anemia (no = 0, yes = 1.333) + NEUT (1.731 × NEUT) + BUN (0.403 × BUN) + ALB (−12.767 × ALB + 70.220) + CRP (1.928 × CRP).
8 Years Survival Probability = (0.000001759 × nomogram score^3^) + (−0.000866931 × nomogram score^2^) + (0.127270769 × nomogram score − 4.952721122);
10 Years Survival Probability = (0.000001759 × nomogram score^3^) + (−0.000826752 × nomogram score^2^) + (0.114371878 × nomogram score − 4.032932803);
15 Years Survival Probability = (0.000001759 × nomogram score^3^) + (−0.000742618 × nomogram score^2^) + (0.089347059 × nomogram score − 2.4122808).

### 3.5. Rationality analysis

The Nomogram score, compared with a single variable, yields a high net benefit and has a better ability to assess the risk of CVD mortality. The DCA clinical benefit interval of the nomogram score of the final model was better than that of any single variable. Among the single variables, age, CKD, and BUN had the best ability to assess the risk of CVD mortality (Fig. S2, Supplemental Digital Content 6).

### 3.6. Segmented survival analysis

Kaplan–Meier (KM) curves were constructed based on the tertiles of the nomogram score. In both the training and validation sets, the KM curve for the high-risk group (T_1_) exhibited the most rapid decline, which corresponded to the lowest survival rate. Significant differences in survival were observed between the high-risk group (T_1_) and both the medium-risk group (T_2_) and low-risk group (T_3_; *P* for log-rank < 0.0001; Fig. 5).

**Figure 5.**
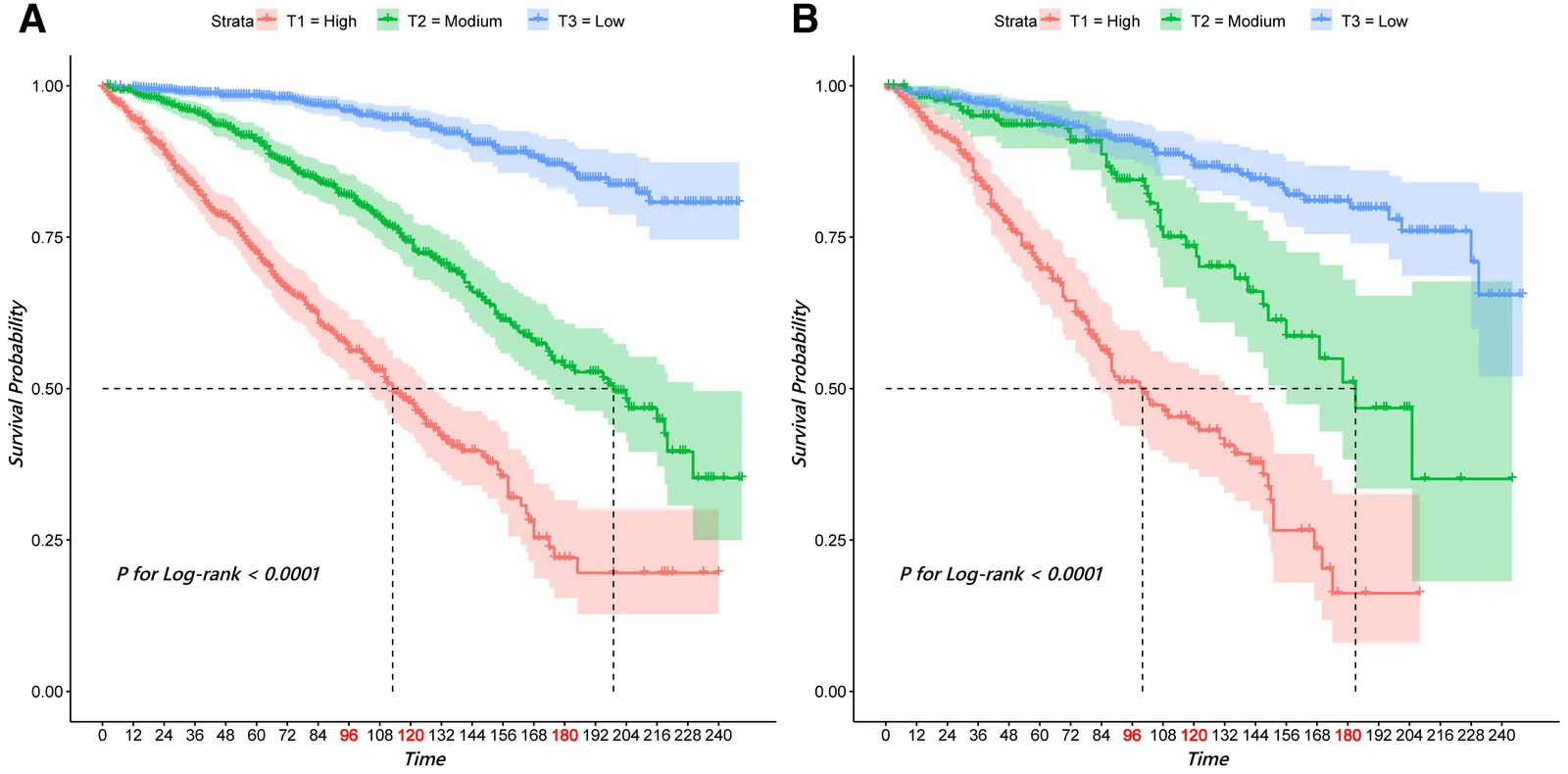
Survival analysis on the training and validation sets. The KM curves of the training set (A) and validation set (B) of the nomogram score in the 3 quartiles, with the horizontal axis representing survival rate and the vertical axis representing time points. Different colors represent the high, medium, and low quartiles of the nomogram score. KM = Kaplan–Meier.

### 3.7. Multiple machine learning models and SHAP features

Decision Tree, Light Gradient Boosting Machine, Naive Bayes, RF Support Vector Machine, and eXtreme Gradient Boosting (XGB) models were used to evaluate the validation set. The parameters were shown in Table S5, Supplemental Digital Content 7. The RF model had the best discrimination (AUC = 0.829, 95% CI: 0.792–0.867), followed by the XGB model (AUC = 0.813, 95% CI: 0.781–0.846; Table 3). To further analyze the decision-making mechanisms of the best models on the validation set, RF and XGB models, we presented and analyzed the distribution of the influence of SHAP features, and showed the prediction results of the case model by superimposing the waterfall plot of the SHAP values of the same participant. In the RF model, age, CKD, and CRP were the most widely distributed and contributed the highest to the prediction of CVD mortality risk, while in the XGB model, age, CRP, and NEUT were the most widely distributed and contributed the highest to the prediction of CVD mortality risk (Figs. 6 and 7).

**Table 3:** Performance evaluation of multiple machine learning models of validation set.

Model	AUC 95% CI	F1 score	Precision	Spe	Sen
DT	0.720 (0.680–0.760)	0.302	0.194	0.805	0.764
LGBM	0.787 (0.752–0.823)	0.547	0.456	0.754	0.683
NB	0.724 (0.686–0.762)	0.510	0.387	0.656	0.744
RF	0.829 (0.792–0.867)	0.631	0.553	0.823	0.746
SVM	0.633 (0.590–0.676)	0.409	0.284	0.436	0.765
XGB	0.813 (0.781–0.846)	0.592	0.508	0.795	0.717

AUC = area under curve, CI = confidence interval, DT = decision tree, LGBM = light gradient boosting machine, NB = Naive Bayes, Neg Pred = negative predicted, Pos Pred = positive predictive, RF = Random Forest, Sen = sensitivity, Spe = specificity, SVM = support vector machine, XGB = extreme gradient boosting.

**Figure 6.**
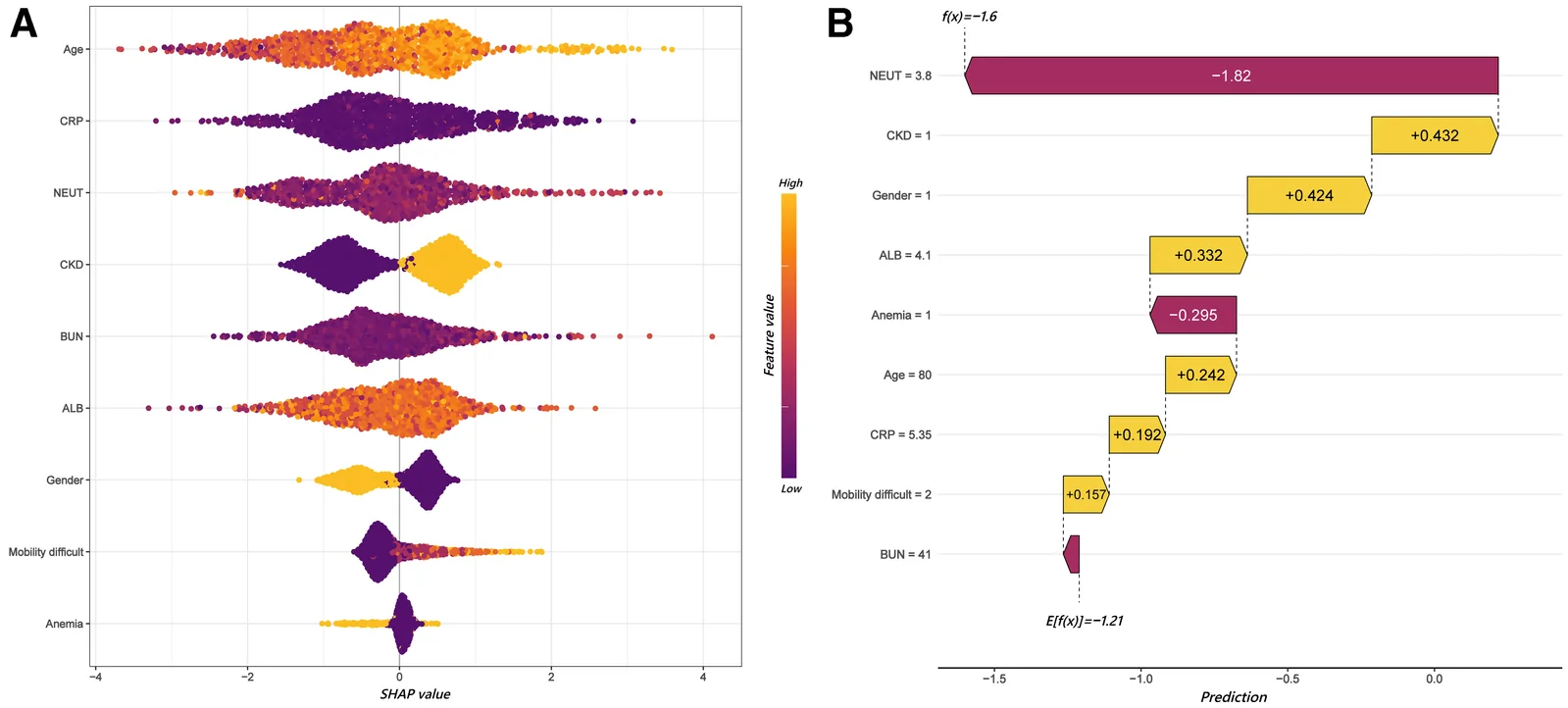
Feature impact and individual prediction of the RF model via SHAP values on the validation set. SHAP feature impact distribution derived from the RF model was presented to quantify and rank contribution of each feature. The wider distribution of SHAP values, more significant impact on prediction outcomes. Positive or negative SHAP contribution value was associated with higher or lower predicted value of target variable (A). SHAP values were visualized in layered manner to illustrate how feature contributions accumulate and ultimately yield prediction result. The *f*(*x*) value positioned at the top was base prediction value, while *E*[*f*(*x*)] value located at the bottom was the expected model contribution (B). ALB = albumin, BUN = blood urea nitrogen, CKD = chronic kidney disease, CRP = C-reactive protein, NEUT = neutrophil, RF = Random Forest, SHAP = SHapley Additive exPlanations.

**Figure 7.**
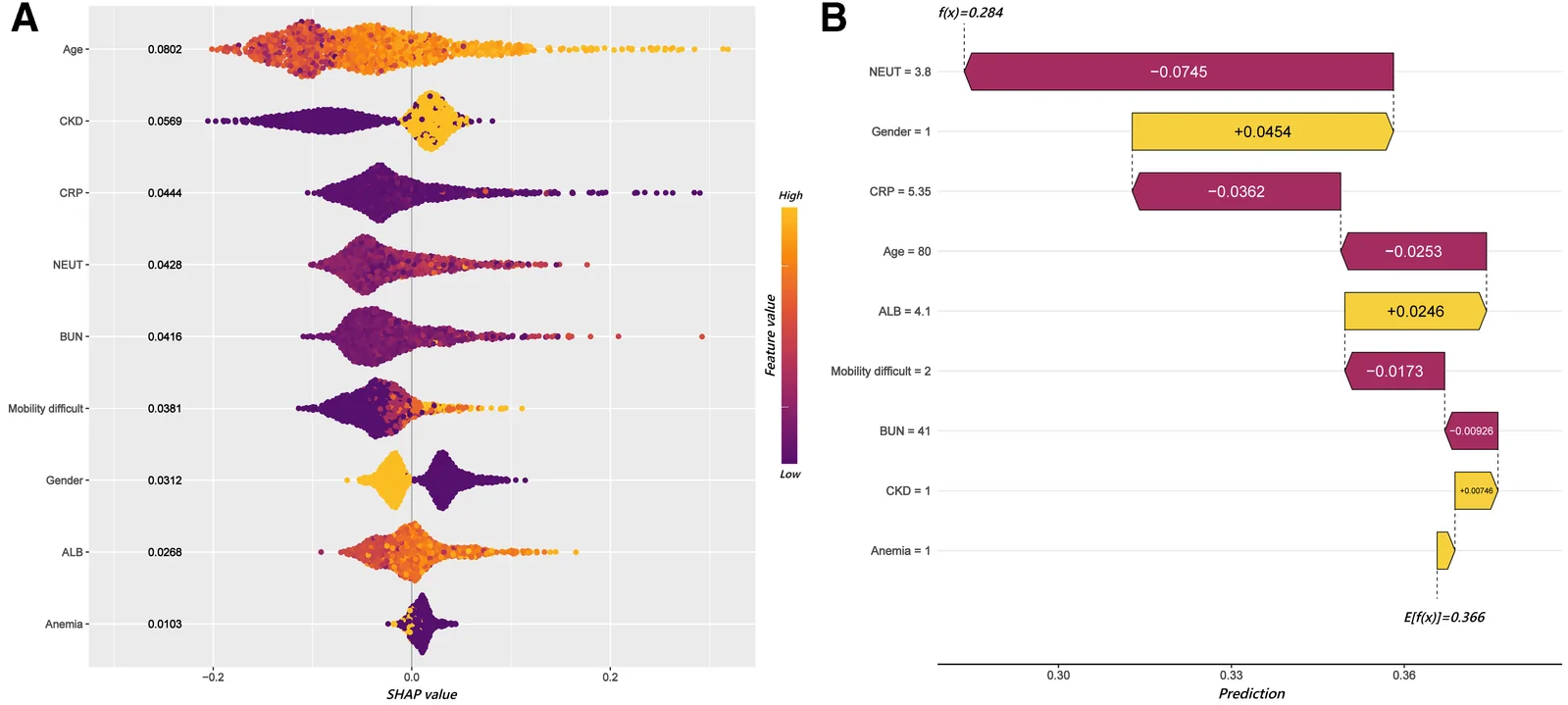
Feature impact and individual prediction of the XGBoost model via SHAP values on the validation set. SHAP feature impact distribution derived from the XGB model was presented to quantify and rank contribution of each feature. The wider distribution of SHAP values, more significant impact on prediction outcomes. Positive or negative SHAP contribution value was associated with higher or lower predicted value of target variable (A). SHAP values were visualized in layered manner to illustrate how feature contributions accumulate and ultimately yield prediction result. The *f*(*x*) value positioned at the top was base prediction value, while *E*[*f*(*x*)] value located at the bottom was the expected model contribution (B). ALB = albumin, BUN = blood urea nitrogen, CKD = chronic kidney disease, CRP = C-reactive protein, NEUT = neutrophil, SHAP = SHapley Additive exPlanations, XGB = extreme gradient boosting.

Data from the training set were presented in Table S6, Supplemental Digital Content 8, Figure S3, Supplemental Digital Content 9, and Figure S4, Supplemental Digital Content 10.

## 4. Discussion

A convenient model for the prediction of CVD mortality in stage 4 CKM was developed and validated by the application of multiple machine learning on NHANES (1999–2018) data. Nine variables: age, sex, CKD, mobility difficulty, anemia, NEUT, BUN, ALB, and CRP were identified as independent predictors of CVD mortality. The resulting model enables accurate risk stratification for CVD mortality and provides a practical decision support tool for clinical practice.

Patients aged ≥60 years frequently present with arteriosclerosis, reduced renal function, and multiple comorbidities, whereby the risk of CVD mortality is markedly increased.^[7]^ In a retrospective cohort of 7456 community-dwelling individuals aged ≥65 years, a positive association between absolute CVD risk score and both CVD mortality and 5-year disability incidence was observed; CVD mortality was estimated to increase by approximately 30% for each additional decade of age.^[26]^ CVD mortality has been reported to be higher among male than among female CKM patients, an observation that has been attributed to androgen-mediated atherogenesis.^[27]^ In a cohort of 2829 patients with early-onset CVD, a significantly elevated all-cause mortality risk was observed in males relative to females (HR = 1.42, 95% CI: 1.04–1.92).^[28]^ Post-menopause, CVD risk in females converges toward that observed in males, reflecting a decline in estrogen-mediated protection. Consequently, the elevated incidence of CVD in postmenopausal females is associated with increased mortality risk.

In addition to renin–angiotensin–aldosterone system overactivity, the principal mechanisms by which CKD precipitates CVD are encompassed by toxin inflammation, oxidative stress, and fibrosis. As renal function declines, phosphate, urea, and other uremic toxins accumulate, thereby triggering systemic inflammation and vascular calcification that culminate in myocardial fibrosis and adverse CVD remodeling. These processes collectively augment cardiac workload and are associated with a markedly elevated incidence of CVD.^[29,30]^ CKD constitutes a potent risk factor for CVD mortality, with a pronounced stepwise increase in risk across CKD stages. In a cross-sectional analysis of 3331 CKD patients, significantly elevated neutrophil-to-albumin ratio was observed among those with stage 3, and high neutrophil-to-albumin ratio was associated with increased CVD mortality (HR = 1.513, 95% CI: 1.131–2.024).^[31]^ CKD patients have been reported to exhibit 2-3-fold higher CVD mortality risk than the general population, with risk escalating in parallel with declining eGFR and accumulating metabolic disturbances (hyperphosphatemia, insulin resistance), indicating that impaired renal function and metabolic dysregulation jointly propel CVD mortality.^[32]^

Physical inactivity is strongly associated with muscle wasting and reduced cardiopulmonary reserve, both of which markedly elevate the risk of CHF and sudden CVD mortality. A 1 metabolic equivalent of task reduction in cardiopulmonary fitness has been associated with a 20% increase in heart failure risk and a 14% increase in the risk of sudden CVD mortality.^[33]^ Sedentary behavior has been linked to elevated CVD risk (HR = 1.34, 95% CI: 1.26–1.43), whereas regular physical activity has been associated with risk reduction (HR = 0.71, 95% CI: 0.66–0.77).^[34]^

Anaemia affects approximately one-third of the global population, with principal etiologies encompassing nutritional deficiencies, chronic infections, and systemic chronic diseases.^[35]^ Evidence indicates that anemia impairs quality of life, and when hemoglobin concentration falls below 11 g/dL, it doubles CVD mortality; this excess risk is most pronounced among individuals with CKD. As renal function declines, indoxyl sulfate and *p*-hydroxyphenylacetic acid accumulate within the bone marrow microenvironment, where erythropoietin synthesis is suppressed and iron metabolism is disrupted. These disturbances impair erythropoiesis, shorten red cell survival, and thereby amplify both anemia and CVD mortality.^[36]^

Increase in the count of NEUTs, an important type of inflammatory cell, often indicates a persistent inflammatory state in the body. Prospective cohort studies showed that when the peripheral NEUT was greater than 7.5 × 10^9^/L, the incidence of CVD in subjects increased significantly. And for every approximately 1.5 × 10^9^/L increase, the risk of ischemic heart disease increased by 15% (OR = 1.15, 95% CI: 1.080–1.210), and the risk of myocardial infarction increased by 22% (OR = 1.22, 95% CI: 1.120–1.340).^[37]^ CRP, an acute response protein, is significantly elevated in inflammatory conditions. CRP levels above 3 mg/L are significantly associated with unstable plaques, acute coronary events, and the risk of sudden death, and it is considered an independent predictor of sudden death.^[38]^

BUN and ALB are important biomarkers for assessing renal function and nutritional status. Levels of BUN above 25 mg/dL typically reflect insufficient renal perfusion and a high catabolic state of protein metabolism, a phenomenon that is particularly prominent in patients with CKD and often indicates a poor prognosis. A cohort study involving 17,719 adults found that the risk of CVD mortality was 48% higher in the high BUN group compared with the low BUN group (HR = 1.48, 95% CI: 1.080–2.020).^[39]^ ALB levels below 3.5 g/dL are often a sign of malnutrition inflammation complex syndrome. A cross-sectional study involving 1070 patients with CVD found that for every 1 g/dL decrease in ALB after adjusting for multiple factors such as age, gender, and comorbidities, the risk of CVD mortality increased by 2.85 times (HR = 3.85, 95% CI: 2.380–6.250).^[40]^

Collectively, these risk factors exert synergistic effects; identification of high-risk phenotypic clusters is essential for early intervention and improved prognosis. Clinical attention should be directed toward surveillance and therapeutic strategies targeting these high-risk cohorts to diminish CVD mortality among patients with CKM syndrome. A user-friendly, web-based calculator was developed to provide graphical estimates of CVD mortality risk at multiple time points. Concurrently, individuals stratified into the high-risk nomogram tier exhibited significantly lower survival probabilities than the low-risk tier. These findings indicate that the prognostic model possesses strong discriminative capacity and may serve as a valuable clinical decision support tool.

Our study provides an assessment of CVD mortality among participants with CKM enrolled in NHANES. Strengths include the large sample size, risk factor selection performed in accordance with strengthening the reporting of observational studies guidelines, and the model’s ready applicability to clinical settings. Several limitations should nevertheless be acknowledged. Comorbidity data were extracted from the database without standardized clinical verification, introducing potential misclassification bias. Furthermore, the absence of repeated measurements precluded trajectory modeling, external validation was not undertaken, and head-to-head comparisons with existing models were not performed, thereby limiting generalizability. Future multicenter investigations incorporating repeated measures and multimodal modeling are therefore warranted.

In our study, risk factors for CVD mortality were evaluated among participants with CKM. A highly generalizable predictive model was subsequently developed, in which the relative contribution of each risk factor is quantified and visually displayed, thereby furnishing clinicians with an evidence-based instrument for risk stratification and for the formulation of preventive and interventional strategies against CVD mortality in CKM patients.

## Acknowledgments

We extend our gratitude to all the participants who contributed to this study.

## Author contributions

**Data curation:** Sining Wang, He Yang, Yixin He, Zhihui Xu, Taijin Wu, Zhouyu Su.

**Supervision:** Weizhe Deng.

**Visualization:** Qiang Zhang.

**Writing – original draft:** Pengyu Zhang, Qiang Zhang.

**Writing – review & editing:** Pengyu Zhang, Qiang Zhang.




















