# Supplementary material for: Machine learning based predictive model for cardiovascular disease mortality risk in patients with stage 4 cardiovascular–kidney–metabolic syndrome
Source: Medicine (Baltimore). 2026 Oct 23;105(43):e51266. doi: 10.1097/MD.0000000000051266 (PMC13706199; doi:10.1097/MD.0000000000051266)
Supplement: Supplementary file 1 [file medi-105-e51266-s001.docx]

**Supplementary Table 1** Presentation of the missing rate of covariates

| Covariates | Frequency | Missing | Covariates | Frequency | Missing |
| --- | --- | --- | --- | --- | --- |
| Gender | 2893 | **0 (0.00)** | SBP Avg | 2745 | **148 (5.12)** |
| Age | 2893 | **0 (0.00)** | DBP Avg | 2745 | **148 (5.12)** |
| BMI | 2760 | **133 (4.60)** | WBC | 2743 | **150 (5.18)** |
| Drink | 2576 | **317 (10.96)** | LYM | 2733 | **160 (5.53)** |
| Smoke | 2892 | **1 (0.03)** | MONO | 2733 | **160 (5.53)** |
| Hypertension | 2893 | **0 (0.00)** | NEUT | 2733 | **160 (5.53)** |
| Diabetes | 2893 | **0 (0.00)** | PLT | 2743 | **150 (5.18)** |
| CHF | 2847 | **46 (1.59)** | RBC | 2743 | **150 (5.18)** |
| CHD | 2828 | **65 (2.25)** | Hb | 2743 | **150 (5.18)** |
| Angina | 2844 | **49 (1.69)** | UACR | 2736 | **157 (5.43)** |
| MI | 2875 | **18 (0.62)** | UPRO | 2736 | **157 (5.43)** |
| Stroke | 2879 | **14 (0.48)** | BUN | 2663 | **230 (7.95)** |
| CVD | 2893 | **0 (0.00)** | UA | 2662 | **231 (7.98)** |
| CKD | 2581 | **312 (10.78)** | Scr | 2678 | **215 (7.43)** |
| CKD stage | 2521 | **372 (12.86)** | eGFR | 2678 | **215 (7.43)** |
| Asthma | 2887 | **6 (0.21)** | ALB | 2680 | **213 (7.36)** |
| COPD | 2871 | **22 (0.76)** | ALT | 2672 | **221 (7.64)** |
| Cancer | 2887 | **6 (0.21)** | AST | 2671 | **222 (7.67)** |
| Sleep disorder | 2111 | **782 (27.03)** | GLB | 2675 | **218 (7.54)** |
| Mobility difficult | 2485 | **408 (14.10)** | GLU | 2679 | **214 (7.40)** |
| Anemia | 2743 | **150 (5.18)** | HbA1c | 2743 | **150 (5.18)** |
| CHD family history | 2761 | **132 (4.56)** | Ca | 2677 | **216 (7.47)** |
| CKM | 2893 | **0 (0.00)** | Fe | 2676 | **217 (7.50)** |
| High GLU | 2679 | **214 (7.40)** | Na | 2680 | **213 (7.36)** |
| Low HDL-C | 2409 | **484 (16.73)** | K | 2680 | **213 (7.36)** |
| High TG | 2653 | **240 (8.30)** | TC | 2682 | **211 (7.29)** |
| Abdominal obesity | 2606 | **287 (9.92)** | TG | 2653 | **240 (8.30)** |
| MetS | 2186 | **707 (24.44)** | LDL | 2525 | **368 (12.72)** |
| CVD mort | 2893 | **0 (0.00)** | HDL | 2682 | **211 (7.29)** |
| Permth exm | 2892 | **0 (0.00)** | CRP | 2039 | **854(29.52)** |
|  |  |  | VitD | 2464 | **429 (14.83)** |

Note: BMI: body mass index, CHF: congestive heart failure, CHD: coronary heart disease, MI: myocardial infarction, CVD: cardiovascular disease, CKD: chronic kidney disease, COPD: chronic obstructive pulmonary disease, CKM: cardiovascular-kidney-metabolic, Mets: metabolic syndrome, SBP Avg: systolic blood pressure average, DBP Avg: diastolic blood pressure average, WBC: white blood cell, LYM: lymphocyte, MONO: monocyte, NEUT: neutrophil, PLT: platelet, RBC: red blood cell, Hb: hemoglobin, UACR: urinary albumin creatinine ratio, UPRO: urinary protein, BUN: blood urea nitrogen, UA: uric acid, Scr: serum creatinine, eGFR: estimated glomerular filtration rate, ALB: albumin, ALT: alanine aminotransferase, AST: aspartate aminotransferase, GLB: globulin, GLU: glucose, HbA1c: glycated hemoglobin, Ca: Calcium, Fe: Iron, Na: Sodium, K: Potassium, TC: total cholesterol, TG: triglyceride, LDL-C: low density lipoprotein cholesterol, HDL-C: high density lipoprotein cholesterol, CRP: C - reactive protein, VitD: Vitamin D.
