# Supplementary material for: Machine learning based predictive model for cardiovascular disease mortality risk in patients with stage 4 cardiovascular–kidney–metabolic syndrome
Source: Medicine (Baltimore). 2026 Oct 23;105(43):e51266. doi: 10.1097/MD.0000000000051266 (PMC13706199; doi:10.1097/MD.0000000000051266)
Supplement: Supplementary file 2 [file medi-105-e51266-s002.docx]

**Supplementary Table 2** Baseline characteristics of participants on training set and validation set

| Characteristics | Total n = 2,893 | Training n = 2,025 (70.00%) | Validation n = 868 (30.00%) | P value |
| --- | --- | --- | --- | --- |
| CVD mortality, n (%) | 659 (22.78%) | 459 (22.67%) | 200 (23.04%) | 0.864 |
| All cause mortality, n (%) | 1,305 (45.11%) | 919 (45.38%) | 386 (44.47%) | 0.681 |
| Survival time, month | 77.00 (38.00, 129.00) | 77.00 (37.00, 128.00) | 78.00 (38.00, 129.00) | 0.792 |
| Age, M (P25, P75) | 69.00 (60.00, 78.00) | 69.00 (60.00, 79.00) | 68.50 (59.00, 77.00) | 0.096 |
| Gender, n (%) |  |  |  | 0.639 |
| Male | 1,619 (55.96%) | 1,127 (55.65%) | 492 (56.68%) |  |
| Female | 1,274 (44.04%) | 898 (44.35%) | 376 (43.32%) |  |
| BMI, M (P25, P75) | 29.00 (25.40, 33.37) | 28.82 (25.40, 33.10) | 29.43 (25.45, 33.75) | 0.152 |
| SBP Avg, M (P25, P75) | 130.67 (117.33, 146.00) | 130.67 (118.00, 146.00) | 130.67 (116.67, 145.33) | 0.478 |
| DBP Avg, M (P25, P75) | 68.00 (60.00, 76.00) | 67.33 (60.00, 75.33) | 69.33 (60.00, 76.67) | 0.140 |
| Drink, n (%) | 1,687 (58.31%) | 1,189 (58.72%) | 498 (57.37%) | 0.529 |
| Smoke, n (%) | 1,735 (59.97%) | 1,193 (58.91%) | 542 (62.44%) | 0.083 |
| Sleep disorder, n (%) | 1,090 (37.68%) | 752 (37.14%) | 338 (38.94%) | 0.381 |
| CHD family history, n (%) | 628 (21.71%) | 426 (21.04%) | 202 (23.27%) | 0.198 |
| Mobility difficult, n (%) |  |  |  | 0.336 |
| No difficult | 1,944 (67.20%) | 1,371 (67.70%) | 573 (66.01%) |  |
| Some difficult | 550 (19.01%) | 370 (18.27%) | 180 (20.74%) |  |
| Much difficult | 237 (8.19%) | 173 (8.54%) | 64 (7.37%) |  |
| Unable | 162 (5.60%) | 111 (5.48%) | 51 (5.88%) |  |
| Complications, n (%) |  |  |  |  |
| Hypertension | 2,077 (71.79%) | 1,458 (72.00%) | 619 (71.31%) | 0.741 |
| Diabetes | 1,244 (43.00%) | 877 (43.31%) | 367 (42.28%) | 0.638 |
| CHF | 873 (30.18%) | 605 (29.88%) | 268 (30.88%) | 0.623 |
| CHD | 1,088 (37.61%) | 764 (37.73%) | 324 (37.33%) | 0.871 |
| Angina | 734 (25.37%) | 520 (25.68%) | 214 (24.65%) | 0.593 |
| MI | 1,159 (40.06%) | 825 (40.74%) | 334 (38.48%) | 0.273 |
| Stroke | 996 (34.43%) | 691 (34.12%) | 305 (35.14%) | 0.629 |
| CKD | 1,351 (46.70%) | 963 (47.56%) | 388 (44.70%) | 0.171 |
| Asthma | 556 (19.22%) | 399 (19.70%) | 157 (18.09%) | 0.337 |
| COPD | 222 (7.67%) | 162 (8.00%) | 60 (6.91%) | 0.352 |
| Cancer | 589 (20.36%) | 422 (20.84%) | 167 (19.24%) | 0.353 |
| Anemia | 473 (16.35%) | 328 (16.20%) | 145 (16.71%) | 0.777 |
| High GLU | 1634 (56.48%) | 1164 (57.48%) | 470 (54.15%) | 0.106 |
| Low HDL | 937 (32.39%) | 653 (32.25%) | 284 (32.72%) | 0.837 |
| High TG | 972 (33.60%) | 686 (33.88%) | 286 (32.95%) | 0.659 |
| Abdominal obesity | 1,991 (68.82%) | 1,396 (68.94%) | 595 (68.55%) | 0.870 |
| MetS | 1,651 (57.07%) | 1,175 (58.02%) | 476 (54.84%) | 0.122 |
| CKD stage |  |  |  | 0.308 |
| Stage I | 168 (5.81%) | 125 (6.17%) | 43 (4.95%) |  |
| Stage II | 314 (10.85%) | 221 (10.91%) | 93 (10.71%) |  |
| Stage III | 720 (24.89%) | 506 (24.99%) | 214 (24.65%) |  |
| Stage IV | 81 (2.80%) | 64 (3.16%) | 17 (1.96%) |  |
| Stage V | 31 (1.07%) | 20 (0.99%) | 11 (1.27%) |  |
| - Laboratory examinations,   M (P25, P75) |  |  |  |  |
| WBC, 10^3^/uL | 6.90 (5.70, 8.20) | 6.90 (5.70, 8.30) | 6.80 (5.70, 8.20) | 0.244 |
| LYM, 10^3^/uL | 1.80 (1.40, 2.30) | 1.80 (1.40, 2.30) | 1.80 (1.40, 2.30) | 0.926 |
| MONO, 10^3^/uL | 0.60 (0.50, 0.70) | 0.60 (0.50, 0.70) | 0.60 (0.40, 0.70) | 0.126 |
| NEUT, 10^3^/uL | 4.20 (3.20, 5.20) | 4.20 (3.30, 5.20) | 4.10 (3.20, 5.20) | 0.126 |
| PLT, 10^3^/uL | 223.00 (187.00, 267.92) | 223.00 (189.00, 268.00) | 224.00 (186.00, 267.00) | 0.510 |
| RBC, 10^6^/uL | 4.58 (4.23, 4.93) | 4.58 (4.24, 4.93) | 4.57 (4.20, 4.95) | 0.620 |
| Hb, g/dL | 13.95 (12.90, 15.00) | 13.97 (12.90, 15.00) | 13.92 (12.90, 15.10) | 0.849 |
| UACR, mg/g | 14.29 (6.62, 46.89) | 14.50 (6.79, 48.92) | 13.82 (6.18, 40.62) | 0.087 |
| UPRO, ug/mL | 15.60 (6.60, 48.76) | 15.60 (6.80, 51.00) | 15.55 (6.30, 46.23) | 0.224 |
| BUN, mg/dL | 16.00 (12.00, 21.00) | 16.00 (12.00, 21.00) | 16.00 (12.00, 21.00) | 0.558 |
| UA, mg/dL | 5.94 (5.00, 7.00) | 6.00 (5.00, 7.00) | 5.90 (5.10, 6.90) | 0.635 |
| Scr, mg/dL | 1.00 (0.80, 1.20) | 1.00 (0.80, 1.20) | 1.00 (0.81, 1.20) | 0.640 |
| eGFR, ml/min | 70.66 (53.52, 88.17) | 70.75 (53.19, 87.98) | 70.46 (54.01, 88.72) | 0.571 |
| ALB, mg/dL | 4.10 (3.90, 4.30) | 4.10 (3.90, 4.30) | 4.10 (3.90, 4.30) | 0.145 |
| ALT, U/L | 20.00 (16.00, 26.00) | 20.00 (16.00, 26.00) | 20.00 (16.00, 26.00) | 0.871 |
| AST, U/L | 23.00 (19.00, 27.53) | 23.00 (19.00, 27.71) | 23.00 (19.00, 27.02) | 0.226 |
| GLB, U/L | 3.00 (2.70, 3.30) | 3.00 (2.70, 3.30) | 3.05 (2.80, 3.30) | 0.054 |
| GLU, mg/dL | 103.00 (93.00, 123.00) | 103.00 (93.00, 123.00) | 103.00 (92.00, 121.92) | 0.201 |
| HbA1c, % | 5.80 (5.50, 6.40) | 5.89 (5.50, 6.45) | 5.80 (5.40, 6.40) | 0.220 |
| Ca, mmol/L | 2.35 (2.28, 2.40) | 2.35 (2.28, 2.40) | 2.35 (2.28, 2.40) | 0.727 |
| Fe, mmol/L | 79.00 (62.00, 99.00) | 80.00 (62.00, 99.57) | 77.07 (60.75, 99.00) | 0.187 |
| Na, mmol/L | 139.55 (138.00, 141.00) | 139.40 (138.00, 141.00) | 139.81 (138.00, 141.00) | 0.211 |
| K, mmol/L | 4.10 (3.90, 4.33) | 4.10 (3.90, 4.33) | 4.10 (3.90, 4.34) | 0.800 |
| TC, mg/dL | 179.00 (154.00, 209.00) | 180.00 (155.00, 210.00) | 175.00 (152.00, 209.00) | 0.092 |
| TG, mg/dL | 125.00 (89.00, 173.00) | 125.00 (89.00, 175.00) | 123.12 (88.00, 168.21) | 0.658 |
| LDL-C, mg/dL | 99.03 (79.00, 124.00) | 100.00 (80.00, 124.00) | 99.00 (78.00, 123.00) | 0.184 |
| HDL-C, mg/dL | 48.00 (40.00, 58.00) | 48.00 (40.00, 58.00) | 48.00 (40.00, 58.00) | 0.952 |
| CRP, mg/dL | 0.32 (0.17, 0.58) | 0.32 (0.17, 0.57) | 0.32 (0.18, 0.62) | 0.480 |
| VitD, nmol/L | 61.10 (45.90, 77.90) | 61.13 (47.00, 78.30) | 61.10 (44.38, 77.50) | 0.261 |

Note: CKM: cardiovascular-kidney-metabolic, CVD: cardiovascular disease, BMI: body mass index, SBP Avg: systolic blood pressure average, DBP Avg: diastolic blood pressure average, CHF: congestive heart failure, CHD: coronary heart disease, MI: myocardial infarction, CKD: chronic kidney disease, COPD: chronic obstructive pulmonary disease, Mets: metabolic syndrome, WBC: white blood cell, LYM: lymphocyte, MONO: monocyte, NEUT: neutrophil, PLT: platelet, RBC: red blood cell, Hb: hemoglobin, UACR: urinary albumin creatinine ratio, UPRO: urinary protein, BUN: blood urea nitrogen, UA: uric acid, Scr: serum creatinine, eGFR: estimated glomerular filtration rate, ALB: albumin, ALT: alanine aminotransferase, AST: aspartate aminotransferase, GLB: globulin, GLU: glucose, HbA1c: glycated hemoglobin, Ca: Calcium, Fe: Iron, Na: Sodium, K: Potassium, TC: total cholesterol, TG: triglyceride, LDL-C: low density lipoprotein cholesterol, HDL-C: high density lipoprotein cholesterol, CRP: C-reactive protein, VitD: Vitamin D.

P < 0.05 indicates a significant difference.
