# Supplementary material for: Machine learning based predictive model for cardiovascular disease mortality risk in patients with stage 4 cardiovascular–kidney–metabolic syndrome
Source: Medicine (Baltimore). 2026 Oct 23;105(43):e51266. doi: 10.1097/MD.0000000000051266 (PMC13706199; doi:10.1097/MD.0000000000051266)
Supplement: Supplementary file 3 [file medi-105-e51266-s003.docx]

**Supplementary Table 3** Baseline characteristics of participants with stage 4 CKM on training set

| Characteristics | Total  n = 2025 | Alive and no-CVD mortality  n = 1566 | CVD mortality  n = 459 | P value |
| --- | --- | --- | --- | --- |
| Age, M (P25, P75) | 69.00 (60.00, 79.00) | 67.00 (58.00, 76.00) | 75.00 (68.00, 80.00) | < 0.001 |
| Gender, n(%) |  |  |  | 0.037 |
| Male | 1127 (55.65) | 852 (54.41) | 275 (59.91) |  |
| Fmale | 898 (44.35) | 714 (45.59) | 184 (40.09) |  |
| BMI, M (P25, P75) | 28.82 (25.40, 33.10) | 29.02 (25.60, 33.50) | 28.10 (24.73, 31.79) | < 0.001 |
| SBP Avg, M (P25, P75) | 130.67 (118.00, 146.00) | 129.33 (117.33, 144.00) | 135.03 (120.00, 153.33) | < 0.001 |
| DBP Avg, M (P25, P75) | 67.33 (60.00, 75.33) | 68.00 (60.67, 76.00) | 65.33 (58.67, 72.67) | < 0.001 |
| Drink, n (%) | 1189 (58.72) | 912 (58.24) | 277 (60.35) | 0.419 |
| Smoke, n(%) | 1193 (58.91) | 930 (59.39) | 263 (57.30) | 0.424 |
| Sleep disorder, n (%) | 752 (37.14) | 612 (39.08) | 140 (30.50) | < 0.001 |
| CHD family history, n (%) | 1599 (78.96) | 1235 (78.86) | 364 (79.30) | 0.839 |
| Mobility difficult, n (%) |  |  |  | 0.004 |
| No difficult | 1371 (67.70) | 1081 (69.03) | 290 (63.18) |  |
| Some difficult | 370 (18.27) | 283 (18.07) | 87 (18.95) |  |
| Much difficult | 173 (8.54) | 131 (8.37) | 42 (9.15) |  |
| Unable | 111 (5.48) | 71 (4.53) | 40 (8.71) |  |
| Complications, n (%) |  |  |  |  |
| Hypertension | 1458 (72.00) | 1120 (71.52) | 338 (73.64) | 0.374 |
| Diabetes | 877 (43.31) | 667 (42.59) | 210 (45.75) | 0.230 |
| CHF | 605 (29.88) | 444 (28.35) | 161 (35.08) | 0.006 |
| CHD | 764 (37.73) | 597 (38.12) | 167 (36.38) | 0.499 |
| Angina | 520 (25.68) | 393 (25.10) | 127 (27.67) | 0.267 |
| MI | 825 (40.74) | 613 (39.14) | 212 (46.19) | 0.007 |
| Stroke | 691 (34.12) | 543 (34.67) | 148 (32.24) | 0.334 |
| CKD | 963 (47.56) | 652 (41.63) | 311 (67.76) | < 0.001 |
| Asthma | 399 (19.70) | 322 (20.56) | 77 (16.78) | 0.073 |
| COPD | 162 (8.00) | 117 (7.47) | 45 (9.80) | 0.105 |
| Anemia | 328 (16.20) | 228 (14.56) | 100 (21.79) | < 0.001 |
| Cancer | 422 (20.84) | 328 (20.95) | 94 (20.48) | 0.829 |
| High GLU | 1164 (57.48) | 894 (57.09) | 270 (58.82) | 0.508 |
| Low HDL | 653 (32.25) | 512 (32.69) | 141 (30.72) | 0.426 |
| High TG | 686 (33.88) | 529 (33.78) | 157 (34.20) | 0.866 |
| Abdominal obesity | 1396 (68.94) | 1095 (69.92) | 301 (65.58) | 0.077 |
| MetS | 1175 (58.02) | 905 (57.79) | 270 (58.82) | 0.693 |
| CKD stage, n (%) |  |  |  | < 0.001 |
| Stage I | 125 (6.17) | 92 (5.87) | 33 (7.19) |  |
| Stage II | 221 (10.91) | 154 (9.83) | 67 (14.60) |  |
| Stage III | 506 (24.99) | 343 (21.90) | 163 (35.51) |  |
| Stage IV | 64 (3.16) | 34 (2.17) | 30 (6.54) |  |
| Stage V | 20 (0.99) | 13 (0.83) | 7 (1.53) |  |
| - Laboratory examinations, - M (P25, P75) |  |  |  |  |
| WBC, 10^3^/uL | 6.90 (5.70, 8.30) | 6.90 (5.70, 8.29) | 7.00 (5.70, 8.40) | 0.514 |
| LYM, 10^3^/uL | 1.80 (1.40, 2.30) | 1.90 (1.50, 2.30) | 1.70 (1.30, 2.10) | < 0.001 |
| MONO, 10^3^/uL | 0.60 (0.50, 0.70) | 0.60 (0.50, 0.70) | 0.60 (0.50, 0.70) | 0.014 |
| NEUT, 10^3^/uL | 4.20 (3.30, 5.20) | 4.17 (3.20, 5.20) | 4.40 (3.40, 5.40) | 0.037 |
| PLT, 10^3^/uL | 223.00 (189.00, 268.00) | 223.76 (189.35, 267.00) | 221.99 (184.50, 271.00) | 0.455 |
| RBC, 10^6^/uL | 4.58 (4.24, 4.93) | 4.59 (4.27, 4.93) | 4.53 (4.13, 4.91) | 0.016 |
| Hb, g/dL | 13.97 (12.90, 15.00) | 14.00 (12.90, 15.00) | 13.80 (12.70, 14.90) | 0.025 |
| UACR, mg/g | 14.50 (6.79, 48.92) | 12.50 (6.36, 38.50) | 26.14 (9.55, 86.41) | < 0.001 |
| UPRO, ug/mL | 15.60 (6.80, 51.00) | 13.85 (6.40, 39.76) | 25.90 (8.40, 88.25) | < 0.001 |
| BUN, mg/dL | 16.00 (12.00, 21.00) | 15.30 (12.00, 20.00) | 18.00 (14.00, 24.00) | < 0.001 |
| UA, mg/dL | 6.00 (5.00, 7.00) | 5.90 (4.90, 6.90) | 6.10 (5.20, 7.30) | < 0.001 |
| Scr, mg/dL | 1.00 (0.80, 1.20) | 0.97 (0.80, 1.20) | 1.06 (0.90, 1.35) | < 0.001 |
| eGFR, ml/min | 70.75 (53.19, 87.98) | 73.95 (57.20, 89.94) | 59.17 (43.68, 79.29) | < 0.001 |
| ALB, mg/dL | 4.10 (3.90, 4.30) | 4.10 (3.90, 4.30) | 4.10 (3.90, 4.30) | 0.302 |
| ALT, U/L | 20.00 (16.00, 26.00) | 20.73 (16.00, 27.00) | 18.00 (15.00, 24.00) | < 0.001 |
| AST, U/L | 23.00 (19.00, 27.71) | 23.00 (19.00, 27.99) | 23.00 (19.00, 27.00) | 0.671 |
| GLB, U/L | 3.00 (2.70, 3.30) | 3.00 (2.70, 3.30) | 3.06 (2.70, 3.40) | 0.015 |
| GLU, mg/dL | 103.00 (93.00, 123.00) | 103.00 (93.00, 122.00) | 104.00 (93.00, 126.00) | 0.354 |
| HbA1c, % | 5.89 (5.50, 6.45) | 5.80 (5.50, 6.40) | 5.90 (5.50, 6.60) | 0.170 |
| Ca, mmol/L | 2.35 (2.27, 2.40) | 2.35 (2.27, 2.40) | 2.35 (2.30, 2.42) | 0.074 |
| Fe, mmol/L | 80.00 (62.00, 99.57) | 80.00 (63.00, 100.00) | 77.00 (61.00, 97.50) | 0.052 |
| Na, mmol/L | 139.40 (138.00, 141.00) | 139.67 (138.00, 141.00) | 139.00 (138.00, 141.00) | 0.030 |
| K, mmol/L | 4.10 (3.90, 4.33) | 4.10 (3.90, 4.30) | 4.17 (3.90, 4.44) | < 0.001 |
| TC, mg/dL | 180.00 (155.00, 210.00) | 179.73 (154.00, 209.00) | 183.00 (158.00, 211.00) | 0.287 |
| TG, mg/dL | 125.00 (89.00, 175.00) | 125.00 (88.00, 174.00) | 125.92 (93.50, 178.00) | 0.260 |
| LDL-C, mg/dL | 100.00 (80.00, 124.00) | 100.00 (80.00, 124.00) | 100.00 (80.00, 124.00) | 0.827 |
| HDL-C, mg/dL | 48.00 (40.00, 58.00) | 48.00 (41.00, 58.00) | 48.00 (40.00, 58.43) | 0.977 |
| CRP, mg/dL | 0.32 (0.17, 0.57) | 0.33 (0.18, 0.56) | 0.31 (0.16, 0.63) | 0.909 |
| VitD, nmol/L | 61.13 (47.00, 78.30) | 61.60 (47.00, 79.83) | 59.60 (47.40, 74.90) | 0.123 |

Note: CKM: cardiovascular-kidney-metabolic, CVD: cardiovascular disease, BMI: body mass index, SBP Avg: systolic blood pressure average, DBP Avg: diastolic blood pressure average, CHF: congestive heart failure, CHD: coronary heart disease, MI: myocardial infarction, CKD: chronic kidney disease, COPD: chronic obstructive pulmonary disease, Mets: metabolic syndrome, WBC: white blood cell, LYM: lymphocyte, MONO: monocyte, NEUT: neutrophil, PLT: platelet, RBC: red blood cell, Hb: hemoglobin, UACR: urinary albumin creatinine ratio, UPRO: urinary protein, BUN: blood urea nitrogen, UA: uric acid, Scr: serum creatinine, eGFR: estimated glomerular filtration rate, ALB: albumin, ALT: alanine aminotransferase, AST: aspartate aminotransferase, GLB: globulin, GLU: glucose, HbA1c: glycated hemoglobin, Ca: Calcium, Fe: Iron, Na: Sodium, K: Potassium, TC: total cholesterol, TG: triglyceride, LDL-C: low density lipoprotein cholesterol, HDL-C: high density lipoprotein cholesterol, CRP: C-reactive protein, VitD: Vitamin D.

P < 0.05 indicates a significant difference.
