# Supplementary material for: Machine learning based predictive model for cardiovascular disease mortality risk in patients with stage 4 cardiovascular–kidney–metabolic syndrome
Source: Medicine (Baltimore). 2026 Oct 23;105(43):e51266. doi: 10.1097/MD.0000000000051266 (PMC13706199; doi:10.1097/MD.0000000000051266)
Supplement: Supplementary file 4 [file medi-105-e51266-s004.docx]

**Supplementary Table 4** Baseline characteristics of participants with stage 4 CKM on validation

| Characteristics | Total  n = 2025 | Alive and no-CVD mortality  n = 1566 | CVD mortality  n = 459 | P value |
| --- | --- | --- | --- | --- |
| Age, M (P25, P75) | 68.50 (59.00, 77.00) | 66.00 (57.00, 74.00) | 76.00 (68.00, 80.00) | < 0.001 |
| Gender, n(%) |  |  |  | 0.017 |
| Male | 492 (56.68) | 364 (54.49) | 128 (64.00) |  |
| Fmale | 376 (43.32) | 304 (45.51) | 72 (36.00) |  |
| BMI, M (P25, P75) | 29.43 (25.45, 33.75) | 29.45 (25.48, 33.99) | 29.35 (25.44, 33.19) | 0.329 |
| SBP Avg, M (P25, P75) | 130.67 (116.67, 145.33) | 129.33 (116.00, 142.67) | 134.25 (119.83, 153.33) | < 0.001 |
| DBP Avg, M (P25, P75) | 69.33 (60.00, 76.67) | 69.33 (60.30, 76.67) | 69.00 (59.33, 76.17) | 0.562 |
| Drink, n (%) | 498 (57.37) | 372 (55.69) | 126 (63.00) | 0.067 |
| Smoke, n(%) | 542 (62.44) | 423 (63.32) | 119 (59.50) | 0.327 |
| Sleep disorder, n (%) | 338 (38.94) | 275 (41.17) | 63 (31.50) | 0.014 |
| CHD family history, n (%) | 666 (76.73) | 507 (75.90) | 159 (79.50) | 0.290 |
| Mobility difficult, n (%) |  |  |  | 0.009 |
| No difficult | 573 (66.01) | 454 (67.96) | 119 (59.50) |  |
| Some difficult | 180 (20.74) | 139 (20.81) | 41 (20.50) |  |
| Much difficult | 64 (7.37) | 44 (6.59) | 20 (10.00) |  |
| Unable | 51 (5.88) | 31 (4.64) | 20 (10.00) |  |
| Complications, n (%) |  |  |  |  |
| Hypertension | 619 (71.31) | 474 (70.96) | 145 (72.50) | 0.672 |
| Diabetes | 367 (42.28) | 279 (41.77) | 88 (44.00) | 0.575 |
| CHF | 268 (30.88) | 194 (29.04) | 74 (37.00) | 0.033 |
| CHD | 324 (37.33) | 246 (36.83) | 78 (39.00) | 0.577 |
| Angina | 214 (24.65) | 159 (23.80) | 55 (27.50) | 0.287 |
| MI | 334 (38.48) | 254 (38.02) | 80 (40.00) | 0.614 |
| Stroke | 305 (35.14) | 221 (33.08) | 84 (42.00) | 0.021 |
| CKD | 388 (44.70) | 252 (37.72) | 136 (68.00) | < 0.001 |
| Asthma | 157 (18.09) | 131 (19.61) | 26 (13.00) | 0.033 |
| COPD | 60 (6.91) | 48 (7.19) | 12 (6.00) | 0.562 |
| Anemia | 145 (16.71) | 105 (15.72) | 40 (20.00) | 0.154 |
| Cancer | 167 (19.24) | 122 (18.26) | 45 (22.50) | 0.182 |
| High GLU | 470 (54.15) | 357 (53.44) | 113 (56.50) | 0.447 |
| Low HDL | 284 (32.72) | 225 (33.68) | 59 (29.50) | 0.269 |
| High TG | 286 (32.95) | 215 (32.19) | 71 (35.50) | 0.382 |
| Abdominal obesity | 595 (68.55) | 459 (68.71) | 136 (68.00) | 0.849 |
| MetS | 476 (54.84) | 362 (54.19) | 114 (57.00) | 0.484 |
| CKD stage, n (%) |  |  |  | < 0.001 |
| Stage I | 43 (4.95) | 30 (4.49) | 13 (6.50) |  |
| Stage II | 93 (10.71) | 60 (8.98) | 33 (16.50) |  |
| Stage III | 214 (24.65) | 137 (20.51) | 77 (38.50) |  |
| Stage IV | 17 (1.96) | 12 (1.80) | 5 (2.50) |  |
| Stage V | 11 (1.27) | 7 (1.05) | 4 (2.00) |  |
| - Laboratory examinations, - M (P25, P75) |  |  |  |  |
| WBC, 10^3^/uL | 6.80 (5.70, 8.20) | 6.80 (5.70, 8.20) | 6.80 (5.70, 8.20) | 0.886 |
| LYM, 10^3^/uL | 1.80 (1.40, 2.30) | 1.80 (1.50, 2.40) | 1.60 (1.30, 2.00) | < 0.001 |
| MONO, 10^3^/uL | 0.60 (0.40, 0.70) | 0.59 (0.40, 0.70) | 0.60 (0.40, 0.70) | 0.660 |
| NEUT, 10^3^/uL | 4.10 (3.20, 5.20) | 4.10 (3.10, 5.03) | 4.30 (3.40, 5.50) | 0.067 |
| PLT, 10^3^/uL | 224.00 (186.00, 267.00) | 225.50 (186.00, 269.00) | 216.50 (184.25, 259.25) | 0.268 |
| RBC, 10^6^/uL | 4.57 (4.20, 4.95) | 4.59 (4.21, 4.95) | 4.49 (4.08, 4.93) | 0.056 |
| Hb, g/dL | 13.92 (12.90, 15.10) | 13.95 (12.90, 15.10) | 13.90 (12.78, 15.10) | 0.968 |
| UACR, mg/g | 13.82 (6.17, 40.62) | 11.87 (5.63, 30.74) | 24.79 (9.36, 72.86) | < 0.001 |
| UPRO, ug/mL | 15.55 (6.30, 46.23) | 13.80 (5.97, 39.18) | 23.25 (10.07, 89.15) | < 0.001 |
| BUN, mg/dL | 16.00 (12.00, 21.00) | 15.00 (12.00, 20.00) | 18.27 (14.00, 24.00) | < 0.001 |
| UA, mg/dL | 5.90 (5.10, 6.90) | 5.90 (5.00, 6.80) | 6.00 (5.20, 7.33) | 0.058 |
| Scr, mg/dL | 1.00 (0.81, 1.20) | 0.98 (0.80, 1.17) | 1.10 (0.90, 1.40) | < 0.001 |
| eGFR, ml/min | 70.46 (54.01, 88.72) | 73.90 (57.85, 90.87) | 58.59 (44.19, 77.75) | < 0.001 |
| ALB, mg/dL | 4.10 (3.90, 4.30) | 4.10 (3.90, 4.30) | 4.10 (3.90, 4.30) | 0.981 |
| ALT, U/L | 20.00 (16.00, 26.00) | 21.00 (16.00, 27.00) | 18.00 (15.00, 24.00) | 0.005 |
| AST, U/L | 23.00 (19.00, 27.02) | 23.00 (19.00, 27.63) | 23.00 (19.00, 27.00) | 0.853 |
| GLB, U/L | 3.05 (2.80, 3.30) | 3.00 (2.80, 3.30) | 3.10 (2.80, 3.34) | 0.092 |
| GLU, mg/dL | 103.00 (92.00, 121.92) | 102.00 (92.00, 120.00) | 105.00 (93.00, 125.25) | 0.167 |
| HbA1c, % | 5.80 (5.40, 6.40) | 5.80 (5.40, 6.40) | 5.80 (5.50, 6.40) | 0.421 |
| Ca, mmol/L | 2.35 (2.28, 2.40) | 2.35 (2.27, 2.40) | 2.35 (2.30, 2.40) | 0.240 |
| Fe, mmol/L | 77.07 (60.75, 99.00) | 77.60 (60.00, 99.00) | 76.00 (62.00, 99.25) | 0.904 |
| Na, mmol/L | 139.81 (138.00, 141.00) | 139.82 (138.00, 141.00) | 139.73 (138.00, 141.00) | 0.860 |
| K, mmol/L | 4.10 (3.90, 4.34) | 4.10 (3.90, 4.33) | 4.20 (3.92, 4.40) | 0.162 |
| TC, mg/dL | 175.00 (152.00, 209.00) | 175.00 (152.72, 206.00) | 173.20 (152.00, 214.25) | 0.852 |
| TG, mg/dL | 123.12 (88.00, 168.21) | 120.00 (86.00, 169.00) | 131.00 (96.89, 167.00) | 0.206 |
| LDL-C, mg/dL | 99.00 (78.00, 123.00) | 99.00 (78.00, 123.00) | 96.25 (74.75, 124.00) | 0.533 |
| HDL-C, mg/dL | 48.00 (40.00, 58.00) | 48.00 (40.00, 57.03) | 49.00 (41.00, 59.00) | 0.397 |
| CRP, mg/dL | 0.32 (0.18, 0.62) | 0.34 (0.19, 0.61) | 0.28 (0.15, 0.68) | 0.155 |
| VitD, nmol/L | 61.10 (44.38, 77.50) | 62.40 (44.70, 79.40) | 57.64 (42.20, 70.42) | 0.005 |

Note: CKM: cardiovascular-kidney-metabolic, CVD: cardiovascular disease, BMI: body mass index, SBP Avg: systolic blood pressure average, DBP Avg: diastolic blood pressure average, CHF: congestive heart failure, CHD: coronary heart disease, MI: myocardial infarction, CKD: chronic kidney disease, COPD: chronic obstructive pulmonary disease, Mets: metabolic syndrome, WBC: white blood cell, LYM: lymphocyte, MONO: monocyte, NEUT: neutrophil, PLT: platelet, RBC: red blood cell, Hb: hemoglobin, UACR: urinary albumin creatinine ratio, UPRO: urinary protein, BUN: blood urea nitrogen, UA: uric acid, Scr: serum creatinine, eGFR: estimated glomerular filtration rate, ALB: albumin, ALT: alanine aminotransferase, AST: aspartate aminotransferase, GLB: globulin, GLU: glucose, HbA1c: glycated hemoglobin, Ca: Calcium, Fe: Iron, Na: Sodium, K: Potassium, TC: total cholesterol, TG: triglyceride, LDL-C: low density lipoprotein cholesterol, HDL-C: high density lipoprotein cholesterol, CRP: C-reactive protein, VitD: Vitamin D.

P < 0.05 indicates a significant difference.
