# Supplementary material for: Machine learning based predictive model for cardiovascular disease mortality risk in patients with stage 4 cardiovascular–kidney–metabolic syndrome
Source: Medicine (Baltimore). 2026 Oct 23;105(43):e51266. doi: 10.1097/MD.0000000000051266 (PMC13706199; doi:10.1097/MD.0000000000051266)
Supplement: Supplementary file 5 [file medi-105-e51266-s007.docx]

**Supplementary Table 5** Parameters of machine learning models

| Model | Function | Parameter | Candidates |
| --- | --- | --- | --- |
| DT | method = "rpart" | cp | 0.01 |
|  |  | maxdepth | 5 |
| LGBM | method = "lightgbm" | n.trees | 500 |
|  |  | nthread | 4 |
|  |  | learning_rate | 0.05 |
|  |  | max_depth | 5 |
| NB | method = "naive_bayes" | usekernel | TRUE |
|  |  | adjust | 0.5 |
| RF | method = "ranger" | mtry | 3 |
|  |  | num.trees | 500 |
| SVM | method = "svmRadial" | C | 1 |
|  |  | kernel | linear |
|  |  | probability | TRUE |
| XGB | method = "xgbTree" | nrounds | 1000 |
|  |  | max_depth | 2 |
|  |  | eta | 0.3 |
|  |  | gamma | 0.001 |
|  |  | colsample_bytree | 0.4 |
|  |  | subsample | 0.7 |

Note: DT: Decision Tree, LGBM: Light Gradient Boosting Machine, NB: Naive Bayes, RF: Random Forest, SVM: Support Vector Machine, XGB: eXtreme Gradient Boosting
