# Supplementary material for: Machine learning based predictive model for cardiovascular disease mortality risk in patients with stage 4 cardiovascular–kidney–metabolic syndrome
Source: Medicine (Baltimore). 2026 Oct 23;105(43):e51266. doi: 10.1097/MD.0000000000051266 (PMC13706199; doi:10.1097/MD.0000000000051266)
Supplement: Supplementary file 6 [file medi-105-e51266-s008.docx]

**Supplementary Table 6** Performance evaluation of multiple machine learning models on training set

| Model | AUC | 95% CI | F1 score | Precision | Specificity | Sensitivity |
| --- | --- | --- | --- | --- | --- | --- |
| DT | 0.660 | 0.641-0.692 | 0.198 | 0.123 | 0.793 | 0.682 |
| LGBM | 0.784 | 0.761-0.806 | 0.527 | 0.415 | 0.682 | 0.752 |
| NB | 0.700 | 0.675-0.725 | 0.455 | 0.332 | 0.524 | 0.802 |
| RF | 0.813 | 0.790-0.837 | 0.577 | 0.493 | 0.791 | 0.703 |
| SVM | 0.669 | 0.641-0.696 | 0.447 | 0.334 | 0.572 | 0.713 |
| XGB | 0.830 | 0.811-0.850 | 0.567 | 0.462 | 0.742 | 0.753 |

Note: DT: Decision Tree, LGBM: Light Gradient Boosting Machine, NB: Naive Bayes, RF: Random Forest, SVM: Support Vector Machine, XGB: eXtreme Gradient Boosting, AUC: area under curve, CI: confidence interval
